# Discerning Quantity: Numerosity in Two Embodied Machine Learning Agents

**DOI:** 10.3390/bs16050813

**Published:** 2026-05-19

**Authors:** Niall Donnelly, Edward Keedwell

**Affiliations:** Faculty of Environment, Science and Economy, University of Exeter, Exeter EX4 4RN, UK

**Keywords:** artificial intelligence, reinforcement learning, numerosity, A-Learning, PPO, Animal-AI, embodiment

## Abstract

As artificial intelligence systems continue to overcome evermore challenging tasks, researchers have suggested that the time is ripe to begin evaluating these systems along more psychologically inspired lines. This study seeks to build upon these recommendations by evaluating two machine learning models, A-Learning and Proximal Policy Optimisation, for the cognitive capability known as numerosity. In our experiment, these two models were embodied in a three-dimensional virtual environment, known as Animal-AI, and tested in a psychologically inspired numerosity experiment. In contrast to previous research, A-Learning failed to reliably express numerosity capabilities, as did Proximal Policy Optimisation. Both models displayed a tendency to overfit to the first policy that provided rewarding feedback. These results suggest that predicting the cognitive capabilities of machine learning models once embodied is non-trivial, and confounding factors such as environmental properties and perceptual processes complicate the expression of numerosity capabilities. Building on these findings, it is suggested that future researchers pay greater attention to the influence of environmental factors and perceptual mechanisms on the machine learning models they are developing, especially if such models are to be embodied in a virtual- or real-world environment.

## 1. Introduction

In recent years, the cognitive capabilities of artificial intelligence (AI) systems have become increasingly comparable to the cognitive capabilities of humans. According to human-centred AI researchers such as Shneiderman (2022) and Capel and Brereton (2023), this has led AI researchers to draw inspiration from the psychological literature to develop research methods for evaluating the latest generation of AI systems. One benefit of this approach is that AI researchers will be able to evaluate the capabilities of AI systems in a more multifaceted, domain-general manner (Hernández-Orallo, 2017; Marcus & Davis, 2020). Here, we seek to build on this trend by assessing the performance of two embodied AI systems on a cognitive capability known as numerosity. To do so, we first discuss recommendations from researchers for evaluating machine learning systems in a domain-general, psychologically inspired manner. We then describe numerosity as a cognitive capability and outline our experiment evaluating two machine learning models, A-Learning and Proximal Policy Optimisation (PPO), in a three-dimensional virtual environment, Animal-AI (AAI). Finally, we discuss the implications of our findings in relation to the broader machine learning literature and offer recommendations for future research.

### 1.1. From Task Assessment to Competency Assessment

According to Mackworth (1993), early AI research was concerned with the capacity of an AI system to classify and manipulate symbols in a rule-based manner. This approach led researchers to adopt various rule-based environments to evaluate the information-processing capabilities of AI systems. Two early examples are chess (Shannon, 1950),and checkers (Samuel, 1959a, 1959b). This trend has continued in recent years with experiments using the more complex game of Go (Silver et al., 2016, 2018, 2017), and in the usage of Atari video game environments (Tsividis et al., 2017) that have the same rules-based properties. Although this approach improved our understanding of the ability of machine learners to optimise performance on a particular objective function (e.g., point accumulation in a game), researchers have criticised this approach for being too task-specific (Hernández-Orallo, 2017), non-generalisable (Hernández-Orallo, 2020), and fragile to environmental modifications (Marcus & Davis, 2020). To address these limitations, researchers have suggested moving towards a more capability-oriented assessment approach (Burnell et al., 2022). To accomplish this, researchers should evaluate AI systems using multiple tests that each investigate a particular aspect of the capability being evaluated. Two capabilities that researchers have proposed, for example, are spatial navigation and object permanence (Burden et al., 2023; Burnell et al., 2023). Based on these suggestions, Voudouris et al. (2024) recommended that AI researchers draw inspiration from comparative psychology practices. This is because comparative psychology research evaluates the cognitive capabilities of various species relative to one another and employs experimental methodologies for this purpose. According to Voudouris et al. (2024), such methodologies might be adapted to compare the cognitive capabilities of AI systems with human and non-human organisms. Voudouris et al. (2022) provides an early example of this approach by comparing human children and AI systems in a series of AAI test batteries. Both the children and the AI systems interacted with the environment using an embodied virtual agent. This enabled Voudouris et al. (2022) to directly compare the performance of the children and the AI systems in the same virtual space, with the same avatar. An advantage of this approach is that Voudouris et al. (2022) compared the children and the AI systems both quantitatively in terms of their scores on various tests and qualitatively by analysing the behaviours and exploration methods expressed by the children and the AI systems. Another benefit of testing embodied AI systems in AAI was demonstrated in research by Cardoso et al. (2023) that tested an embodied machine learner in a series of stimulus-response experiments. These experiments were inspired by research on stimulus-response discrimination in pigeons by Mondragón and Hall (2015). Using pigeons as a baseline, Cardoso et al. (2023) acquired a more informed understanding of the strengths and limitations of the AI system compared to an animal species capable of passing the experiment. This approach aligns with recommendations from other researchers such as Burden et al. (2023), Burnell et al. (2022), Hernández-Orallo (2017, 2020), Marcus and Davis (2020), and Rutar et al. (2025) to evaluate machine learning systems in a more holistic, domain-general manner rather than the more traditional task-optimisation approach. Here, we seek to provide an initial investigation into the utility of these recommendations compared to the methodologies and techniques utilised in earlier AI research. Numerosity is the cognitive capability that was chosen for our experiment. This is because in order to successfully demonstrate numerosity capabilities, an agent must be capable of effectively perceiving, identifying, and discriminating objects of varying quantities in an environment. All of these factors will be influenced by the embodiment process, making numerosity a particularly suitable candidate for comparing early AI approaches with the more recent recommendations. Furthermore, pre-existing research by Lind and Vinken (2021) predicts that A-Learning will have numerosity capabilities in AAI. However, Lind and Vinken (2021) made this prediction using a virtual environment known as the Animal Learning Simulator. This simulator is a rules-based textual environment and suffers from many of the limitations of early AI research. Comparing the results of our experiment with those of Lind and Vinken (2021) therefore enables us to compare recent research recommendations with more traditional research methods. By comparing these approaches, we may better understand the advantages and disadvantages of both.

### 1.2. Numerosity Defined

Perhaps the first description of numerosity as a cognitive capability was provided by Stanley Stevens (Stevens, 1939, p. 1), who defined numerosity as a ‘property defined by certain operations performed upon groups of objects.’ Although Stephen’s definition was accepted by some researchers, such as Call et al. (2017) and Thomas and Chase (1980), it was only partially adopted by the wider academic community. Instead, Stevens definition has been used interchangeably with other terms such as numerousness and numerosity perception (Schmidt, 2022). More recently, numerosity has been defined as ‘the estimation of number—or of its approximation’ (Kluth & Zetzsche, 2016, p. 1), and ‘the perception of number’ (Anobile et al., 2021, p. 1). Consistent amongst all these definitions of numerosity is the capacity to successfully discriminate groups of varying quantity from one another. This is the aspect of numerosity that we seek to evaluate here in embodied A-Learning and PPO agents. Specifically, the capacity of the agents to reliably discriminate groups of varying reward quantity from one another to obtain the greater reward signal from the larger group. Rather than simply reinforcing a policy for obtaining a reward signal of any quantity.

### 1.3. Numerosity Research

One of the first numerosity experiments was carried out by Wolfe and Kaplon (1941), who found that a kernel of corn separated into four pieces would more strongly reinforce novel behaviours in chickens than a single whole piece. This finding suggests that chickens not only respond to the amount of rewarding feedback (that is, corn) provided to them but also take into consideration the number of units the reward is separated into. Since Wolfe and Kaplon (1941)’s experiment, researchers have evaluated numerous animal species at both the behavioural and neurobiological levels for numerosity capabilities. These animal species include human and non-human apes, monkeys, rats, pigeons, crows, songbirds, dogs, cats, frogs, and zebrafish, (Brannon, 2005; Cicchini et al., 2016; Nieder, 2021). Building on this research, numerosity capabilities have also been studied in machine learning systems such as convolutional (Upadhyay & Varma, 2023) and deep (Zhang & Wu, 2020) neural networks. Deep neural networks have produced particularly promising findings. Research by Testolin et al. (2020), for example, found that deep neural networks successfully discriminated quantity in two separate visual stimuli. These stimuli consisted of white dots placed on a black background. Modifications to these visual stimuli (e.g., size and distance of the dots from one another) led to similar variations in performance between deep neural networks and human participants. This finding suggests that deep neural networks have the potential to develop numerosity capabilities that emulate those of humans. According to Testolin et al. (2020), this finding suggests that numerosity capabilities can develop from non-numerical features and challenge suggestions by previous researchers such as Dehaene-Lambertz and Spelke (2015), Feigenson et al. (2004) and Leibovich et al. (2017) that there is a dedicated neuronal mechanism for numerosity capabilities. Similar numerosity capabilities in deep neural networks were also reported by Sabathiel et al. (2020, 2022), where deep neural networks successfully counted the number of stimuli in a grid-based visual input of black-and-white squares. However, both of these experiments tested the capabilities of deep neural networks via two-dimensional visual inputs rather than through embodiment in a more complex three-dimensional space. In addressing this limitation, Pecyna et al. (2022) found that a deep neural network embodied in a robotic avatar successfully counted the number of white ping-pong balls placed on top of a black tablecloth. To achieve this, the robotic avatar provided camera inputs to the deep neural network alongside a robotic arm which pointed at each ping-pong ball in turn. Unlike previous animal experiments, however, the numerosity capabilities demonstrated in these experiments are limited to counting, with no object interactions or perception of object value being required. Animals, for example, are required to not only count the number of objects (e.g., seeds, food pellets, etc.) but also identify these objects as desirable and navigate toward them in order to receive rewarding feedback (e.g., satiation and taste). These capabilities were not required in the aforementioned research using deep neural networks (Pecyna et al., 2022; Sabathiel et al., 2020, 2022; Testolin et al., 2020). Our experiment builds on these findings by requiring the agent to discriminate quantity and interact with rewarding objects. This approach, therefore, provides a closer emulation of numerosity experiments in human and non-human animals.

### 1.4. Current Research

Here, we seek to investigate the influence that embodiment has on the numerosity capabilities of two machine learning systems. Specifically, A-Learning and PPO. A-Learning, developed by Ghirlanda et al. (2020) was selected for our experiment as it has been predicted by Lind and Vinken (2021) to reliably learn numerosity capabilities. As (Lind & Vinken, 2021, p. 15) states:

‘In general, associative learning is expected to result in preferences for the largest quantities of food as long different quantities can be told apart, and A-learning will given sufficient information result in optimal behavior’

However, a limitation of Lind and Vinken (2021)’s experiment is that the experimental design was overly reductive. The A-Learning model was tested in the Animal Learning Simulator, where it had to navigate between several predefined state spaces of varying reward sizes. This experimental design reduces the cognitive complexity of resource management and self-restraint to state-space navigation. As (Lind & Vinken, 2021, p. 11) describes:

‘This was a two-choice task and the following behaviors were included: take the dish with fewer pieces of food or take the dish with more food’

This approach is characteristic of approaches in early AI research and does not align with the aforementioned recommendations by researchers such as Burden et al. (2023), Burnell et al. (2023), Hernández-Orallo (2017, 2020), Marcus and Davis (2020), and Rutar et al. (2025). In line with these recommendations, we aim to test the prediction by Lind and Vinken (2021) that A-Learning is capable of reliably demonstrating numerosity capabilities. To do this, we embody the A-Learning model as an agent in the AAI environment. Based on previous Animal-AI research (Cardoso et al., 2023; Lucas & Prémont-Schwarz, 2024; Voudouris et al., 2022), we predict that A-Learning will not develop numerosity capabilities as reliably as Lind and Vinken (2021) predicts. This finding will likely result from the greater environmental complexity of AAI and, by extension, the greater likelihood that A-Learning will reinforce a policy that seeks a local rather than a global minimum (Sutton & Barto, 2018). In addition to A-Learning, we also tested PPO as a second reinforcement learning agent due to its frequent use in AAI experiments, high performance compared to other machine learning agents, and open access training and testing scripts (Beyret et al., 2019; Crosby et al., 2019, 2020; Voudouris et al., 2025). The performance of PPO serves as an additional benchmark to measure the performance of A-Learning against another machine learning agent that has shown promising results in previous AAI experiments (Crosby et al., 2019, 2020).

## 2. Materials and Methods

### 2.1. Animal-AI

**Figure 1:**
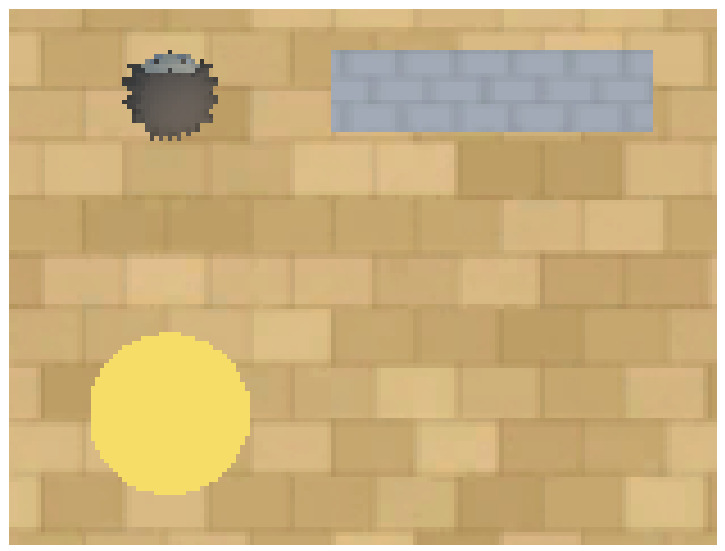
A bird’s eye view of AAI (version 3.0.2) with the materials used to construct the training and testing environments. On the top left is the virtual avatar in which the machine learners are embodied. On the top right is the ‘wall’ asset used to restrict the space in which the agent can navigate. In the bottom left is the reward object, which provides a positive reward signal upon contact with the virtual avatar.

AAI is a virtual environment built on the Unity video game platform (Version: 3.6.1.0, San Francisco, CA, USA) (Juliani et al., 2018) to create purpose-built experiments to evaluate the capabilities of machine learners embodied in a 40 × 40 unit space. The assets for creating experiments in AAI are inspired by the cognitive, comparative, and developmental psychology literature (Beyret et al., 2019; Voudouris et al., 2025). Figure 1 provides an overview of the assets used in AAI to carry out the experiments. To encourage reward-seeking and emulate biological organisms’ hunger mechanisms, AAI provides linear negative feedback as the number of timesteps increases in an episode.

### 2.2. A-Learning and PPO

#### 2.2.1. A-Learning

A-Learning is a reinforcement learning model developed by Ghirlanda et al. (2020) that seeks to incorporate principles from the associative learning literature into a reinforcement learning model. To accomplish this, Ghirlanda et al. (2020) proposes two equations for the weighing and updating of the values of perceived stimuli, and one equation to map stimulus-response values to the probability of expressing various behaviours. We will now describe each of these equations in turn.

The first equation defines how the weighting *w* assigned to a stimulus *s* is modified following exposure to another subsequent stimulus $s^{\prime }$.

(1) $$▵w\left(s\right)=\alpha_{w}\left[u\left(s^{\prime }\right)+w\left(s^{\prime }\right)-w\left(s\right)\right]$$

where ▵w(s) is the change in w(s) as experience is gained. $\alpha_{w}$ serves as a positive learning rate that regulates the magnitude of stimulus updates (Sutton & Barto, 2018). $u(s^{\prime })$ represents the initial predetermined value of s’. This equation enables an agent to modify stimulus weightings in response to experiential learning.

The second equation defines how the value *v* assigned to a stimulus-response pairing s→b is modified.

(2) $$▵v\left(s\to b\right)=\alpha_{v}\left[u\left(s^{\prime }\right)+w\left(s^{\prime }\right)-v\left(s\to b\right)\right]$$

where $\alpha_{v}$ is a second positive learning rate (Sutton & Barto, 2018). Inclusion of the value of the most recent stimulus input w(s’) formalises the assumption that conditioned and unconditioned stimuli are capable of reinforcing behaviour in the same manner. The experience $s\to b\to s^{\prime }$ updates only v(s→b) for an expressed *b*. This formalises an agent updating the value assigned to a conditioning stimulus both when the stimulus is correctly and incorrectly predicted to be present.

The third equation maps the value of a stimulus-response association to the probability of a specific response being expressed Pr(s→b). This equation balances the agent performing exploratory and exploitative behaviours (Sutton & Barto, 2018):

(3) $$Pr\left(s\to b\right)=\frac{e^{\beta v(s\to b)}}{\sum b^{\prime }\;e^{\beta v(s\to b^{\prime })}}$$

Considering all possible behaviours would provide the sum of this equation. The temperature β>0 balances the expression of exploratory and exploitative actions by the agent. A higher value of β results in more exploratory behaviours being expressed, whilst a lower value of β results in more deterministic, reward-greedy behaviours being expressed (Sutton & Barto, 2018). Six variations of these hyperparameter weightings were tested in the first experiment to assess the impact that this would have on performance. These hyperparameter weightings were based on previous hyperparameter weightings used by Lind and Vinken (2021) and Cardoso et al. (2023) and recommendations by Dorigo et al. (1996). Here, the A-Learning model is a Python (Version: 3.12.8) class object created by Cardoso et al. (2023). In order to replicate previous A-Learning research by Cardoso et al. (2023) and Lind and Vinken (2021), the model was tested without prior training.

#### 2.2.2. PPO

PPO is a policy gradient method developed by Schulman et al. (2017) that receives inputs from the environment and, using stochastic gradient ascent, optimises a surrogate objective function to develop an effective policy to interact with the environment. A PPO policy π with parameters θ is updated at each iteration *k* via Equation (4):

(4) $$\theta_{k+1}=\underset{\theta }{argmax}\;\underset{s,a\approx \pi_{\theta_{k}}}{E}\left[L\left(s,a,\theta_{k},\theta \right)\right]$$

where *E* is the expected return for an action *a* performed in a given state *s*. *L* is the return between the state action pair at the current $\theta_{k}$ and previous θ parameter weightings. When $L(s,a,\theta_{k},\theta )$ is positive, the value of the state action pair increases according to Equation (5). When $L(s,a,\theta_{k},\theta )$ is negative, the value of the state action pair decreases according to Equation (6):

(5) $$L\left(s,a,\theta_{k},\theta \right)=min(\frac{\pi_{\theta }\left(a|s\right)}{\pi_{\theta_{k}}\left(a|s\right)},\left(1+\epsilon \right)A^{\pi_{\theta_{k}}})$$

where $A^{\pi_{\theta_{k}}}$ is the current advantage function for θ and ϵ regulates the magnitude with which state action pair values are updated. This equation increases the likelihood of the PPO policy expressing the same state-action pair in future timesteps and episodes. The existence of the min term inhibits the magnitude by which the value of these state action pairs increases. This is to ensure that updates to π are stabilised during training.

(6) $$L\left(s,a,\theta_{k},\theta \right)=max(\frac{\pi_{\theta }\left(a|s\right)}{\pi_{\theta_{k}}\left(a|s\right)},\left(1-\epsilon \right)A^{\pi_{\theta_{k}}})$$

This equation decreases the likelihood of the PPO policy expressing the same state action pair in future timesteps and episodes. The existence of the max term inhibits the magnitude by which the value of these state action pairs decreases. This is to ensure that updates to π are stabilised during training.

**Figure 2:**
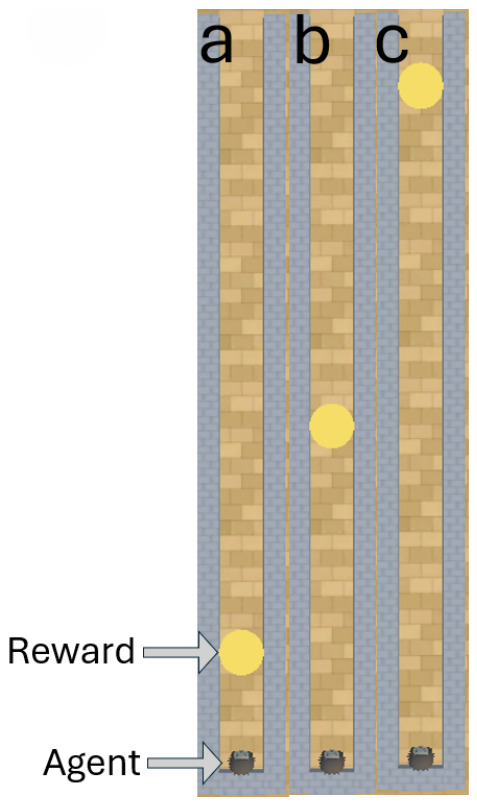
The reward tunnel for creating a trained version of PPO, across 3 episodes labeled (**a**–**c**). As demonstrated, the reward begins at various locations in the tunnel during training. This is to vary how far the agent must navigate to receive rewarding feedback. The reward tunnel is a 2 × 35 unit space, with the agent beginning each episode at the beginning of the tunnel facing the reward. Here, the reward begins 5 (**a**), 15 (**b**) and 30 (**c**) units away from the agent.

**Figure 3:**
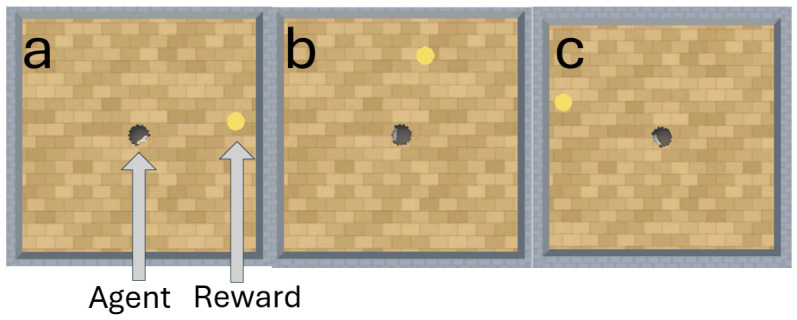
The open field for creating a trained version of PPO, across 3 episodes labelled (**a**–**c**). As demonstrated, the reward begins at various locations and orientations to the agent during training. This is to vary the search behaviours that the agent must express to receive rewarding feedback across episodes. The open field is a 10 × 10 unit space, with the agent beginning each episode in the centre of the field at a random orientation.

**Figure 4:**
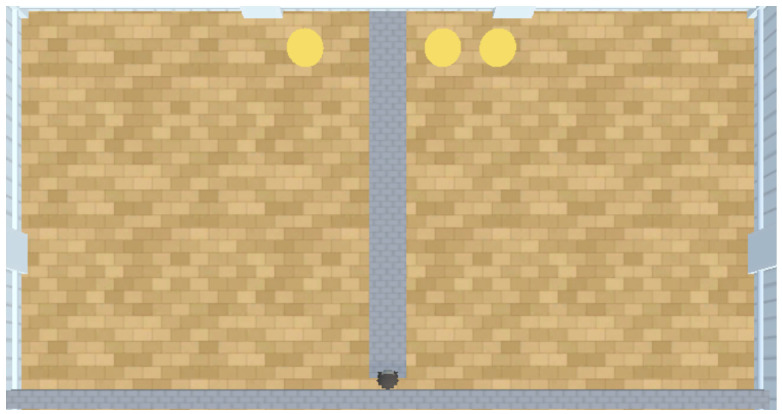
The initial experimental layout from experiment 1. The agent begins each episode on the top of a dividing barrier. This restricts the agent such that only one of the two pits can be entered in each episode. In this layout, the right-hand pit contains the larger quantity of rewards. The agent begins at coordinates (20,1,20), with the rewards at coordinates (15.5,0,38), (23,0,38), and (26,0,38).

In this experiment, the PPO model is a Python class object imported from an online repository known as stable baselines 3 (Version: 1.4.0 https://github.com/DLR-RM/stable-baselines3, accessed on 18 November 2023) (Raffin et al., 2021). According to the findings of Crosby et al. (2019), Johnson and Weitzenfeld (2025), and Voudouris et al. (2025), PPO struggles to perform well in embodiment experiments without prior training. We therefore tested trained and untrained versions of PPO to assess the impact that training has on PPO’s performance. Both A-Learning and the untrained version of PPO do not have experience of interacting with rewards prior to testing. By contrast, trained versions of PPO learn that rewards provide a positive reward signal upon contact prior to testing. Two versions of PPO were trained for our experiments. The first of these PPO agents was embodied in a ‘reward tunnel’ (see Figure 2). At the beginning of each episode, PPO will detect a reward directly ahead. Over numerous episodes, PPO explores the reward tunnel, discovers the reward, and develops a policy to obtain the reward again in later episodes. A potential benefit of being trained in the reward tunnel is that the agent is encouraged to move toward the reward once it is detected. However, it is also possible that policies developed in the reward tunnel may discourage the agent from moving in alternative directions and exploring the wider test space. Therefore, a second PPO agent was embodied in an ‘open field’ (see Figure 3) training environment. In the open field, the reward begins each episode at a random location and orientation to the agent. Over numerous episodes, PPO begins to explore the training space and develop a policy to seek out and obtain the reward. Both agents were trained for 10,000 timesteps prior to testing. Although a richer training curriculum with additional variations (e.g., multiple paths, varying tunnel widths and field sizes, etc.) may have provided a more diverse training experience, we restricted the training layouts to this design. This decision was made to limit the number of factors when comparing the trained PPO’s performance to A-Learning and the untrained version of PPO. In both environments, five versions of PPO with two varied hyperparameter weightings were trained to assess the impact this would have on performance. These two hyperparameters were the learning rate (lr), which regulates the magnitude with which policy and value functions are updated during each timestep, and an entropy coefficient (ec) that serves as a temperature hyperparameter to balance the expression of exploratory and exploitative actions (Sutton & Barto, 2018). Each of the hyperparameter weightings was based on previous research by Lehuger and Crosby (2021) and AAI experiments by Beyret et al. (2019) and Donnelly (2025). A potential confounding factor of the reward tunnel environment is the distance between the reward and the agent at the beginning of each episode. If, for example, the reward is always given close to the agent, PPO may develop a policy that is biased against out-of-distribution cases where rewards are given farther away than in training. It is also possible that if the rewards begin too far away from the agent, an insufficient number of reward interactions may be experienced prior to testing. To address this, two sets of PPO agents with matching hyperparameter weightings were trained in the reward tunnel and tested against each other in the initial layout of experiment 1 (see Figure 4). One set of five PPO agents experienced rewards that were up to 10 units away from the agent, whilst the other set of five experienced rewards that were up to 30 units away. The former set of PPO agents produced an average reward size of 0.26, with a standard deviation of 0.01. The latter set of PPO agents produced an average reward size of 0.52, with a standard deviation of 0.01; outperforming the former set (t(18)=50.97, p<0.0001). Due to this greater performance, the results for the PPO agents trained in the reward tunnel hereafter are the results of the set of PPO agents trained to experience rewards that were up to 30 units away. The untrained version of PPO serves as a baseline performance without prior training. The untrained PPO and A-Learning agents, therefore, serve as direct comparisons of performance with one another.

#### 2.2.3. Heuristic and Random Agents

Two embodied agents were used as control conditions for the experiment. These were a heuristic-based agent (hereafter called the ‘heuristic agent’) and a random action agent (hereafter called the ‘random agent’). The heuristic agent serves as a representation of direct-path performance and is hard-coded to move directly towards rewarding objects when detected. This agent is embedded with simple numerosity capabilities in the form of a behaviour tree. If the heuristic agent detects a larger number of rewards on either side, it will begin to reorient itself towards that direction. In addition to serving as a representation of direct-path performance, the heuristic agent also provides a sanity check for each experiment. If, for example, the heuristic agent successfully passes a numerosity test, then this suggests that the machine learning agents are provided with sufficient sensory inputs to also pass the test. Failure to pass the test by a machine learning agent will therefore likely be due to a lack of numerosity capabilities rather than a scarcity of sensory inputs. The random agent serves as a baseline for chance performance by moving in one of nine directions (forward, backwards, left, right, forward-left, forward-right, backwards-left, backwards-right, or staying stationary) for ten timesteps before randomly selecting another action. All agents were able to move in the same manner.

### 2.3. Method

**Figure 5:**
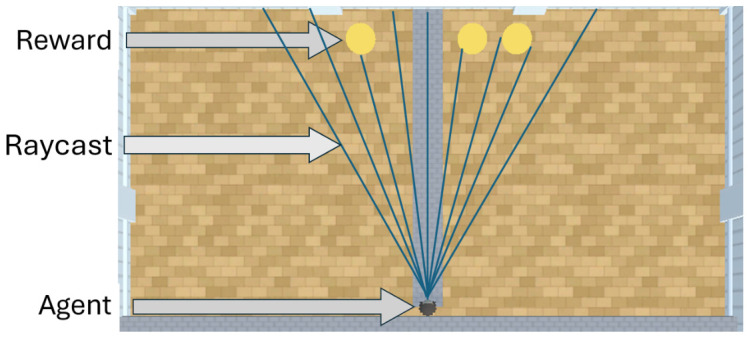
Figure 4 with a visual rendering of the raycasts when 9 raycasts are utilised. The three leftmost rays provide inputs of stimuli to the left of the agent, providing information that there is a reward to the left of the agent. The three middle rays provide inputs of stimuli ahead of the agent, providing information that there is a reward ahead of the agent. The three rightmost rays provide inputs of stimuli to the right of the agent, providing information that there is a reward to the right of the agent.

The A-Learning, PPO, and heuristic agents were embodied in a virtual avatar that is capable of simple sensing and navigation within AAI. This environment was a numerosity experiment that emulated the experimental layout described by Lind and Vinken (2021) in a 40 × 20 unit space (see Figure 4). This experimental layout is also similar to the numerosity tests in the Animal-AI competition (Crosby et al., 2019) from which Lind and Vinken (2021) took inspiration. To sense the environment, the avatar used raycasting to receive sensory inputs. Raycasting works similarly to sonar by emitting ’rays’ into the environment and returning the label of an intersected object (either a reward, a wall, or the boundaries of the arena). The avatar emitted 9 raycasts in a 60-degree range in each experiment. The returned labels from these 9 raycasts were then parsed to identify the objects closest to the agent on the left, right, and straight ahead. The distances of these objects from the agent are also provided in spatial units. A second version of the avatar that emitted 15 raycasts over a 100-degree range was also tested in experiments 2 and 3 due to the larger number and greater spacing of rewards to be detected. These variations may be referred to as the ‘9 ray’ and ‘15 ray’ versions, respectively. At the beginning of each episode, the agent begins on top of a dividing barrier and must enter a pit on either the left- or right-hand side based on the rewards that are detected. As demonstrated in Figure 5 from experiment 1, the agent receives inputs of rewards to the left, ahead, and right of the agent. The agents are therefore required to learn that navigating toward the reward on the left results in one reward being obtained. Navigating toward the reward that is ahead or to the right, however, can result in two rewards being obtained. According to predictions from Lind and Vinken (2021), A-Learning should learn through exploration over several episodes to favour the larger reward option. Here, A-Learning is predicted to learn to favour the rewards that are in the right-hand pit.

#### 2.3.1. Experimental Design

During testing, the agent always began at the same location within the environment. Each reward provides positive feedback at a value of +2. Agents receive a negative reward signal at each timestep of $\frac{-1}{t}$ where *t* is the number of timesteps in an episode (Beyret et al., 2019). Therefore, agents are incentivised to develop time-efficient policies for obtaining the largest quantity of rewards possible. The number of timesteps remaining in an episode is reset when a reward is obtained. This is to ensure that agents are given sufficient time to obtain multiple rewards; failure to do so will be due to an ineffective policy rather than a lack of timesteps. To pass the test, the agent must learn to reliably identify the pit with the greater number of rewards and collect all rewards within it to receive the largest possible reward signal. The environment is a forced-choice environment, where a dividing barrier ensures that agents can enter only one pit per episode. All agents completed 10 iterations of the experiment. An iteration consisted of 1000 episodes, with each episode lasting for 300 timesteps. The A-Learning and PPO agents completed 10 iterations at each hyperparameter weighting, creating a population of 10 ‘individuals’ for each agent. Between-groups effect sizes were calculated using a one-way analysis of variance (Heiman, 2001). Subsequently, pairwise comparisons between agents were performed using a series of independent *t*-tests (Mishra et al., 2019). The performance of the agents was considered significantly different if p≤0.005 after Bonferroni adjustment. The mean reward value obtained by the agent’s performance was visualised in matplotlib version 3.8.1. The experiments were carried out using a Microsoft HP Envy laptop with an i7-1165G7 processor and 16.0 GB of RAM (HP Inc., London, UK). All code used is available upon request to the corresponding author.

#### 2.3.2. Experiment 1

**Figure 6:**
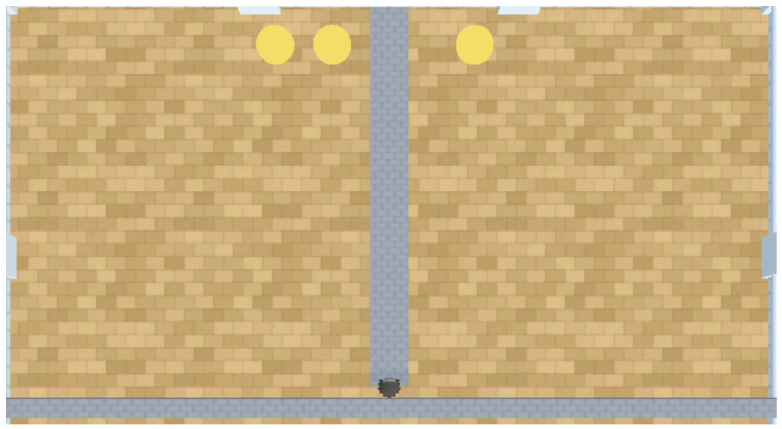
The second test environment of experiment 1 with the larger quantity of rewards in the left-hand pit.The agent begins at coordinates (20,1,20), with the rewards at coordinates (14,0,38), (17,0,38) and (24.5,0,38).

The first experiment consisted of a ‘1v2’ experimental layout (see Figure 4). In this experiment, the 9 ray agents must successfully discriminate between a pit with 1 reward and a pit with 2 rewards. This experiment serves as a base measure using the minimum number of rewards necessary for a numerosity experiment. This experimental layout was used to compare various hyperparameter weightings of A-Learning and PPO against one another. The highest performing A-Learning and PPO hyperparameter weightings were then tested a second time with the left-hand pit containing the larger quantity of rewards (see Figure 6). If an agent develops a policy that is biased to one pit over another, then the agent should perform significantly better when that pit contains the larger quantity of rewards. If no bias exists, then the agent should perform comparably well regardless of which pit contains the larger quantity of reward.

#### 2.3.3. Experiment 2

**Figure 7:**
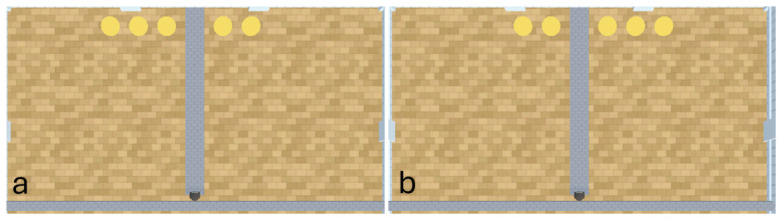
The second experiment layouts. Figure (**a**) displays a 3 left, 2 right, and figure (**b**) displays a 2 left, 3 right reward layout. In figure (**a**) the agent begins at coordinates (20,1,20), with the rewards at coordinates (11,0,38), (14,0,38), (17,0,38), (23,0,38) and (26,0,38). In figure (**b**) the agent begins at coordinates (20,1,20), with the rewards at coordinates (14,0,38), (17,0,38), (23,0,38), (26,0,38), and (29,0,38).

The second experiment consisted of a ‘2v3’ experimental layout (see Figure 7). Here, agents with the highest-performing hyperparameter weightings from experiment 1 must successfully discriminate between a pit with 2 rewards and a pit with 3 rewards. Experiment 2 builds on experiment 1 by requiring the agents to discriminate a larger quantity of rewards from each other. The smaller difference in the reward ratio (i.e., 1:2 in experiment 1 and 1:1.5 in experiment 2) also requires the agents to perform more precise quantity discriminations than in the previous experiment. The 15-ray versions of the A-Learning and PPO agents were tested in addition to the 9-ray versions from experiment 1. This is because there are 5 total rewards in experiment 2, which cannot all be simultaneously detected by the 9 ray agents. By comparison, the 15-ray agents will be capable of simultaneously detecting and responding to all 5 rewards. It is still possible that the 9-ray agents perform well in experiment 2, as the agents may still detect and respond to each of the 5 rewards over numerous timesteps, as is predicted to occur with the heuristic agent, for example. However, the 15-ray versions of A-Learning and PPO will be in an advantageous position, as policy actions can be learnt in response to all 5 rewards in the environment being simultaneously detected. Numerosity capabilities, therefore, are likely to be observed if the 15-ray agents outperform the 9-ray agents.

#### 2.3.4. Experiment 3

**Figure 8:**
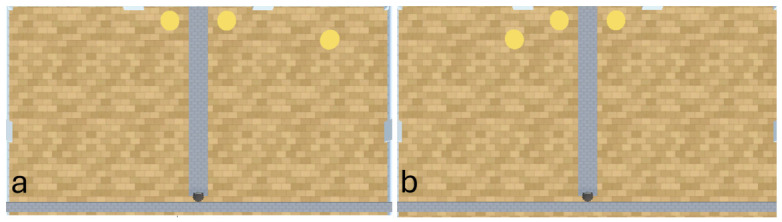
The ‘1v1 (+1 random) experimental layout. Panel (**a**) displays the random reward in the left pit, and panel (**b**) displays the random reward in the right pit. The random reward begins each episode at a random position along the x-axis, except at the diving barrier, where it cannot be placed. The agent begins each episode at coordinates (20,1,20), with the rewards beginning at (17,0,38), (23,0,38) and (−1,0,36), where −1 results in a random value between 1 and 40 (excluding 19 to 21 for the dividing barrier) being generated.

**Figure 9:**
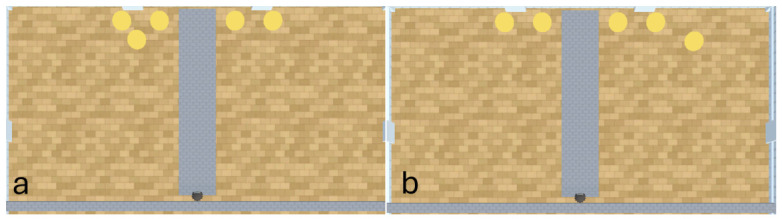
The ‘2v2 (+1 random) experimental layout. Panel (**a**) displays the random reward in the left pit, and panel (**b**) displays the random reward in the right pit. In this layout, the dividing barrier is wider to provide the agents with additional space to search for the random reward before entering a pit. The random reward begins each episode at a random position along the x-axis, except at the diving barrier, where it cannot be placed. The agent begins each episode at coordinates (20,1,20), with the rewards beginning at (12,0,38), (16,0,38), (24,0,38), (28,0,38) and (−1, 0, 36), where −1 results in a random value between 1 and 40 (excluding 18 to 22 for the dividing barrier) being generated.

In experiments 1 and 2, the agents and rewards begin each episode at the same coordinates. It is therefore possible (with fortuitous exploration) that the agents reinforce a sequence of actions to obtain the larger quantity of rewards via route tracking. If developed into a reliable policy, this would give the impression of numerosity capabilities without requiring discrimination of quantity. Experiment 3, therefore, builds on experiments 1 and 2 by introducing additional stochasticity into the environment using a ‘1v1 (+1 random)’ (see Figure 8) and ‘2v2 (+1 random)’ (see Figure 9) experimental layout. The additional noise introduced into this experiment is likely to inhibit the performance of the A-Learning and PPO agents. This is because A-Learning and PPO will be required to search a greater number of spaces in which the random reward may have appeared, increasing the number of timesteps required to correctly identify the pit with more rewards inside. The performance of the heuristic agent is also likely to be inhibited, as the agent might not detect random rewards that are located far away (e.g., Figure 8a) before selecting actions from the behaviour tree. It is also possible for the random reward to occlude other rewards at the beginning of an episode (e.g., Figure 9a). This introduces additional difficulty to the experiment, requiring the agents to detect rewards from several locations to confirm which pit contains the greater quantity of rewards.

## 3. Results

### 3.1. Experiment 1

**Figure 10:**
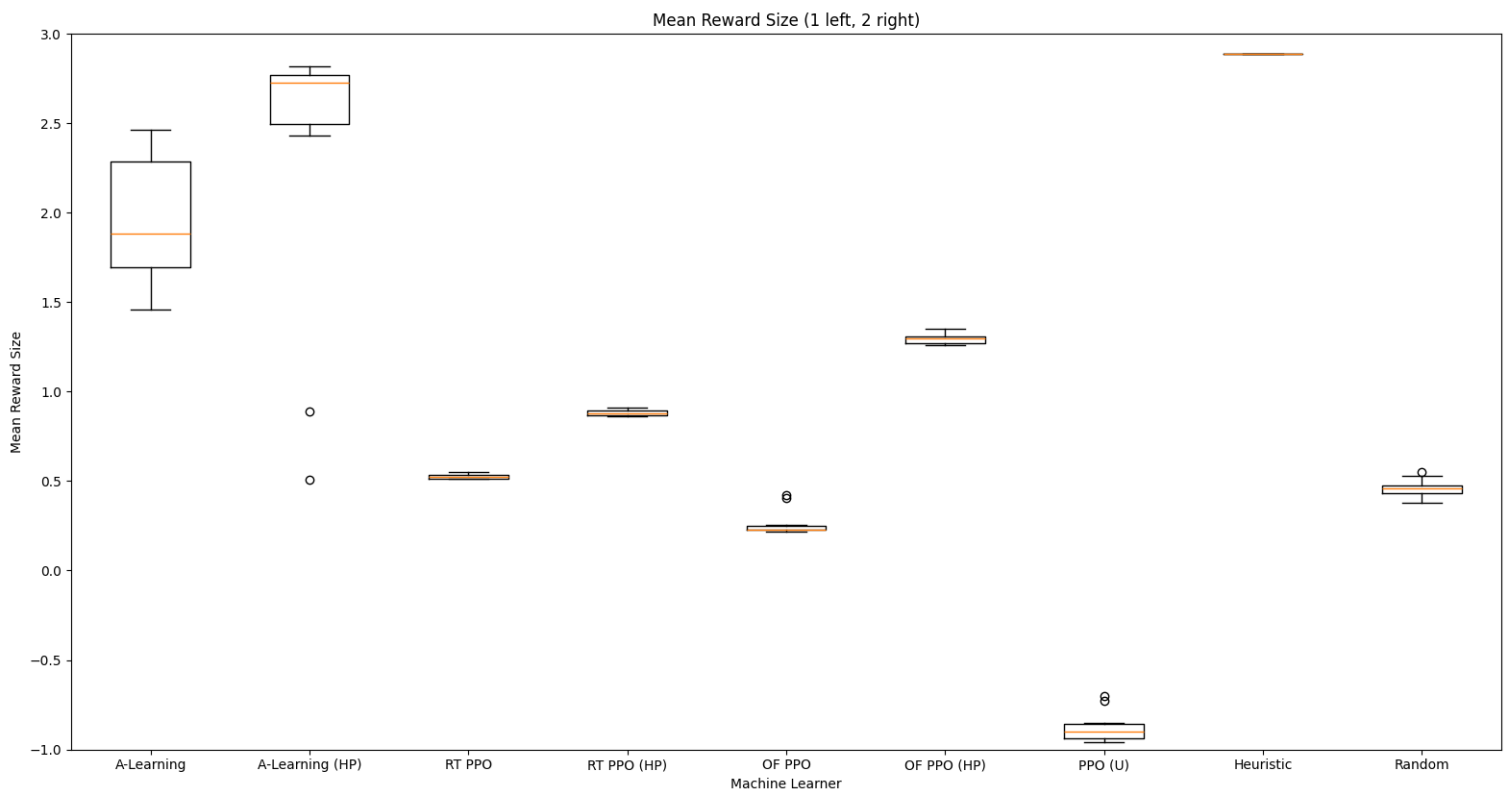
Average reward size obtained for each agent in the 1 left, 2 right experimental layout. Hyperparameter weightings for the highest-performing (HP) A-learning, reward-tunnel (RT) and open-field (OF) PPO agents are the same as the highest-performing hyperparameter weightings from Table 1. Results for the untrained PPO (PPO-U), heuristic, and random action agents are also provided.

**Figure 11:**
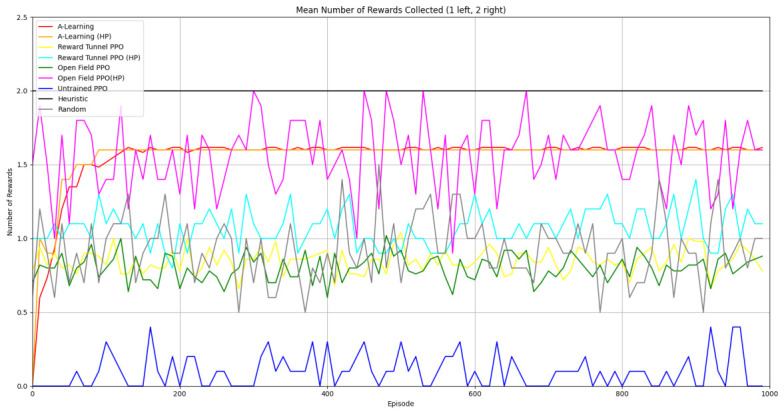
Average number of rewards obtained at each episode in the 1 left, 2 right experimental layout. Hyperparameter weightings for the highest-performing (HP) A-learning, reward-tunnel and open-field PPO agents are the same as the highest-performing hyperparameter weightings from Table 1. Results for the untrained PPO, heuristic, and random action agents are also provided.

**Figure 12:**
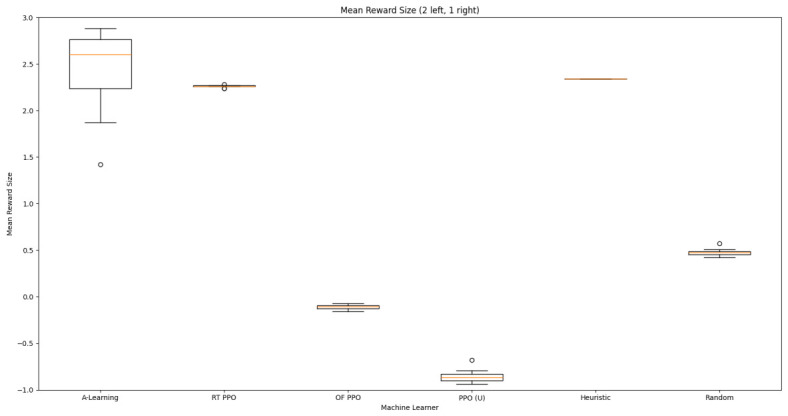
Average reward size obtained for each agent in the 2 left, 1 right experimental layout. Hyperparameter weightings for the highest-performing (HP) A-learning, reward-tunnel (RT) and open-field (OF) PPO agents are the same as the highest-performing hyperparameter weightings from Table 1. Results for the untrained PPO (PPO-U), heuristic, and random action agents are also provided.

**Figure 13:**
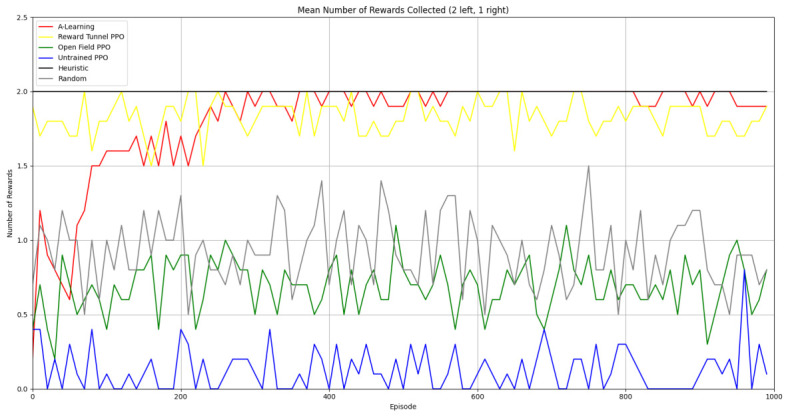
Average number of rewards obtained at each episode in the 2 left, 1 right experimental layout. Hyperparameter weightings for the highest-performing (HP) A-learning, reward-tunnel (RT) and open-field (OF) PPO agents are the same as the highest-performing hyperparameter weightings from Table 1. Results for the untrained PPO (PPO-U), heuristic, and random action agents are also provided.

Reliability in selecting the larger reward option was not observed in reinforcement learning agents during the initial experimental layout. Although a statistically significant difference was observed between agents (F(5,54)=689.67,p<0.0001) and the highest-performing hyperparameter weighting of A-Learning reliably obtained the highest reward amount, this was not observed on average (see Figure 10 and Table 1). A-Learning did obtain a higher average reward value than the reward tunnel (t(18)=12.89,p<0.0001), open field (t(18)=15.54,p<0.0001) and untrained (t(18)=24.72,p<0.0001) PPO agents. The average performance of A-Learning was also higher than the highest-performing hyperparameter weighting of the reward tunnel (t(18)=5.25,p=0.0005) and open field (t(18)=3.73,p=0.005) PPO agents. Suggesting that A-Learning developed a more rewarding policy than any of the PPO agents. This policy was also more stable, as the average number of rewards collected by A-Learning plateaued during testing, whilst PPO remained stochastic (see Figure 11). To test for bias in pit selection, the A-Learning and PPO agents with the highest-performing hyperparameter weightings were tested in a second experimental layout with the number of rewards in each pit swapped (see Figure 6 and Figure 12). A statistically significant difference between models was again observed (F(5,54)=518.69,p<0.0001). Bias for one pit over another was not observed in A-Learning (t(18)=0.39,p=0.70) or untrained PPO (t(18)=0.59,p=0.56) but was observed for PPO agents trained in the reward tunnel (t(18)=242.56,p<0.0001) and open field (t(18)=113.38,p<0.0001). The reward-tunnel PPO was biased toward the left-hand pit, whereas the open-field PPO was biased toward the right-hand pit. In the second experimental layout, A-Learning again outperformed the open field (t(18)=16.73,p<0.0001) and untrained PPO agents (t(18)=21.38,p<0.0001), but not the reward-tunnel PPO agent (t(18)=1.16,p=0.28). The high average reward in both experimental layouts suggests that A-Learning may be capable of developing a policy that selects the larger reward option at a particular hyperparameter weighting. The average number of rewards collected by A-Learning across testing was also more stable than PPO (see Figure 13). The large standard deviation in Table 1 and Table 2 however, suggests that this policy may not emerge reliably in every iteration of testing. The heuristic agent obtained 2 rewards in 100% of episodes for both experimental layouts. The lower average reward obtained by the heuristic agent in the second experimental layout indicates that a greater number of timesteps was required to obtain both rewards. This is despite the rewards being equidistant from the agent in each experimental layout. The behaviour tree on which the heuristic agent operates may therefore be faster at reacting to larger quantities of rewards on the right-hand side as opposed to the left-hand side.

#### 3.1.1. A-Learning

**Figure 14:**
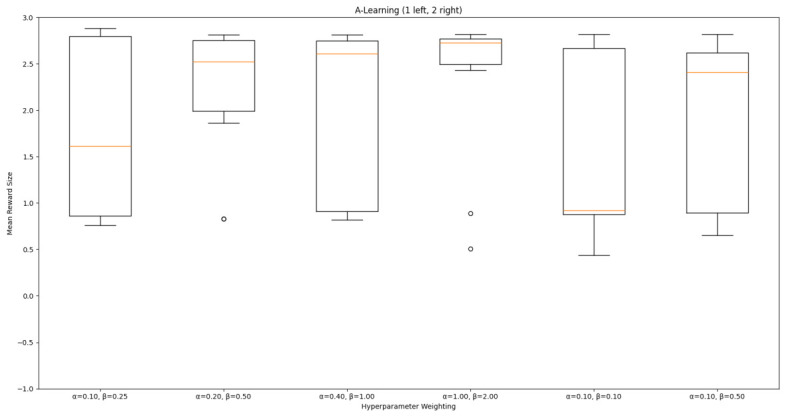
Average reward size obtained by A-Learning at each hyperparameter weighting.

**Figure 15:**
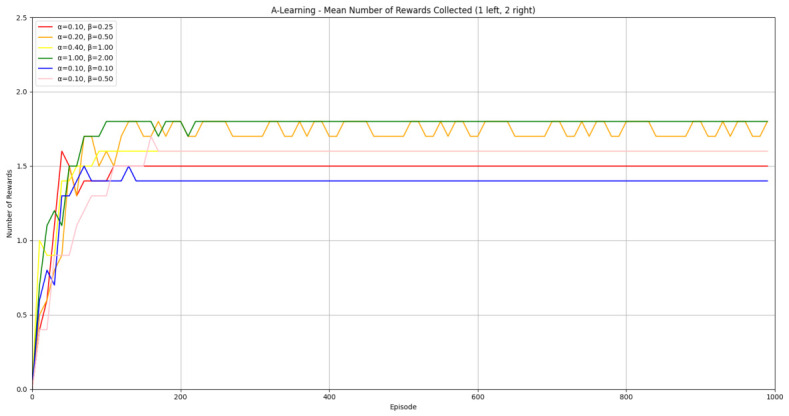
Average number of rewards obtained by A-Learning at each hyperparameter weighting.

**Figure 16:**
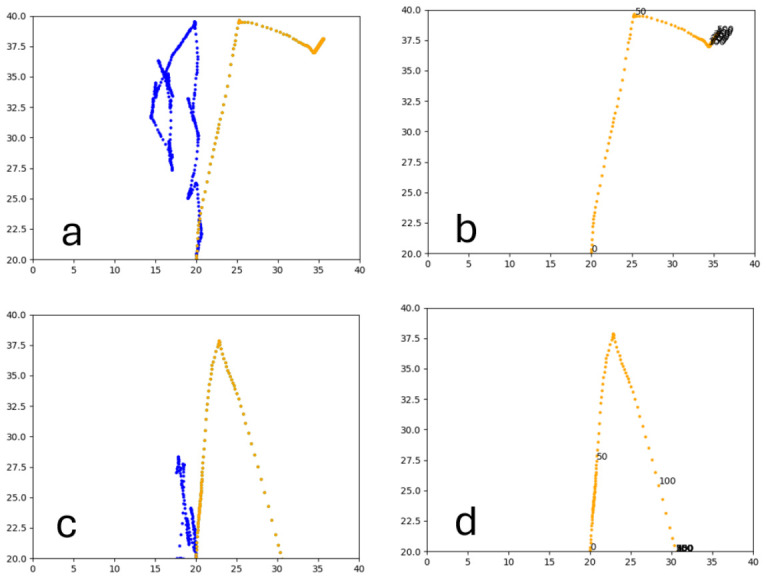
Learning to enter the right-hand pit did not guarantee that A-Learning would successfully collect both rewards. Panel (**a**) shows the movement path of A-Learning at episodes 10 (blue), 100 (green), and 1000 (orange) when successfully developing a policy to obtain both rewards in the right-hand pit. Panel (**b**) plots the movement path of the same policy developed by A-Learning in panel (**a**) at episode 1000, with every 10th timestep numbered. Panel (**c**) shows the movement path of A-Learning at episodes 10 (blue), 100 (green), and 1000 (orange) when obtaining only one of the two rewards in the right-hand pit. The movement path at episode 100 is not visible due to the movement path at episode 1000 overlapping the figure points. Panel (**d**) plots the movement path of the same policy developed by A-Learning in panel (**c**) at episode 1000, with every 50th timestep numbered.

In the initial experiment, A-Learning did not reliably learn to choose the larger reward option (right-hand pit), with the exception of α=1.0,β=2.0, which learnt to acquire two rewards in 8 of the 10 iterations. This unreliability can be observed in the standard deviation at each hyperparameter weighting (see Figure 14, and Table 3). This finding is the result of a tendency of A-Learning to overfit to the initial policy that provides rewarding feedback. In early episodes, A-Learning would explore the environment and, upon discovering one of the rewards, reinforce the sequence of actions that led to obtaining that initial reward. This sequence of actions would continue to be reinforced as these actions are repeated in subsequent episodes. If, for example, A-Learning initially discovered the reward in the left-hand pit, then a policy would be reinforced where the left-hand pit was entered in subsequent episodes. Although the right-hand pit would occasionally be entered as a result of exploratory behaviours (see Equation (3)), this was not sufficient to alter overfitting to the left-hand pit. This overfitting can be observed in the average number of rewards collected during testing (see Figure 15), which plateaued at every hyperparameter weighting. Even if A-Learning entered the right-hand pit first, this did not guarantee that a policy to obtain both rewards would develop. Instead, a policy that reinforced obtaining only one of the two rewards could develop (see Figure 16). The exception is α=1.0,β=2.0, which has the largest α and β weightings of any A-Learning agents tested (see Figure 14 and Table 3). This resulted in more exploratory actions and larger stimulus–behaviour updates than the other A-Learning agents (see Equations (2) and (3)). This, in turn, facilitated more opportunities for discovering the larger reward quantity by chance exploration and more heavily weighing these encounters in stimulus–behaviour updates. Although it may be tempting to suggest that increasing the α and β weightings would further improve performance, a point of instability will be reached when the α and β weightings are so large as to cause unstable updates to the agent’s policy weighting (Ghirlanda et al., 2020; Sutton & Barto, 2018). Therefore, it can be suggested that while A-Learning has the capacity to display numerosity capabilities in an embodied space, this is likely to be the result of sensitive hyperparameter weightings and fortuitous exploration rather than deliberate planning or numerosity capabilities.

#### 3.1.2. Trained PPO

**Figure 17:**
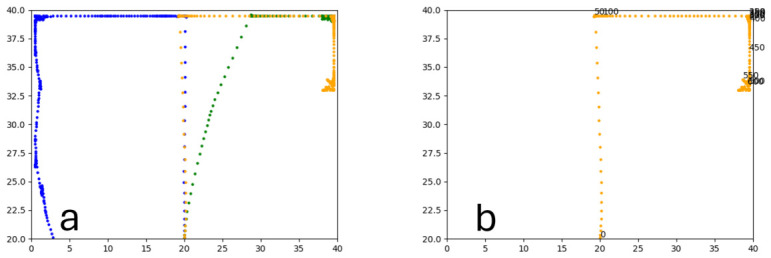
An overview of an effective policy expressed by PPO in the 1 left, 2 right reward layout when trained in the reward tunnel. Panel (**a**) displays the agent entering the less rewarding left-hand pit in early episodes, such as episode 10 (blue). By episode 100 (green), the agent has explored the more rewarding right-hand pit. By the end of testing, the agent has updated its policy to enter the right-hand pit, as shown in episode 1000 (orange). Panel (**b**) displays the movement path at episode 1000, with every 50th timestep numbered.

**Figure 18:**
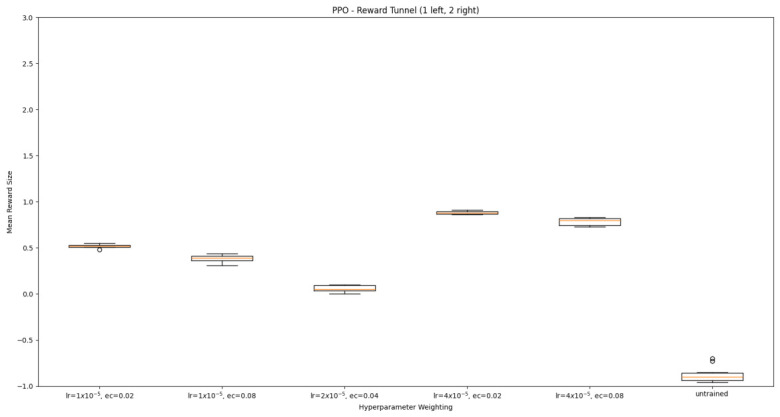
Average reward size obtained by reward-tunnel-trained PPO at each hyperparameter weighting. The mean value acquired by each agent is represented by an orange line.

**Figure 19:**
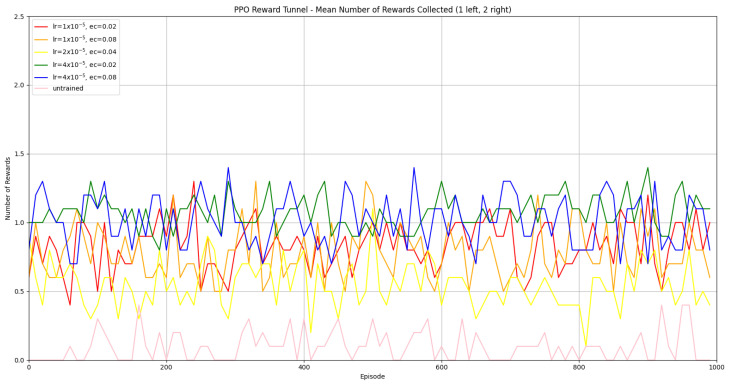
Average number of rewards obtained by reward-tunnel-trained PPO at each hyperparameter weighting.

**Figure 20:**
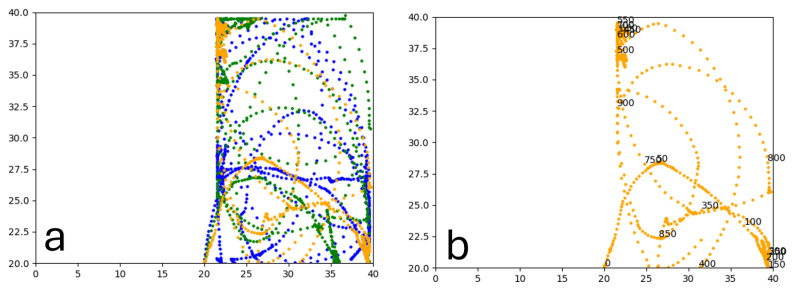
An overview of the policy expressed by PPO in the 1 left, 2 right reward layout when trained in the open field. Panel (**a**) displays how the policy learned in the open field is expressed in early episodes by moving in an arching manner. Episode 10 (blue) and 100 (green) demonstrate this arching pattern. By episode 1000 (orange), the agent’s movement is more focused around the location of the 2 rewards. However, the agent does not reliably obtain 2 rewards during testing. Panel (**b**) displays the movement path at episode 1000, with every 50th timestep numbered.

**Figure 21:**
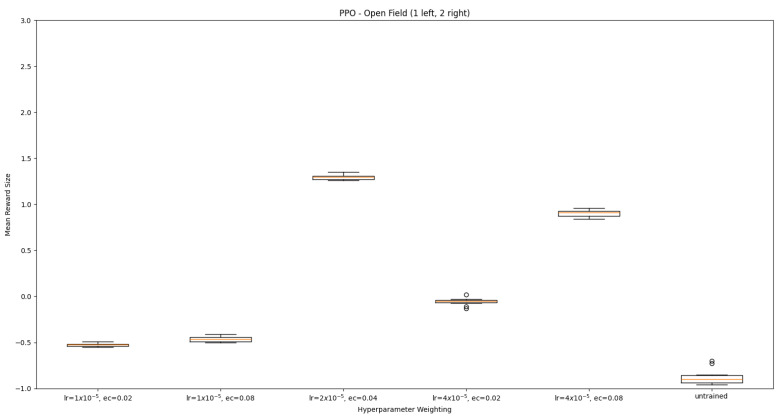
Average reward size obtained by open-field-trained PPO at each hyperparameter weighting. The mean value acquired by each agent is represented by an orange line.

**Figure 22:**
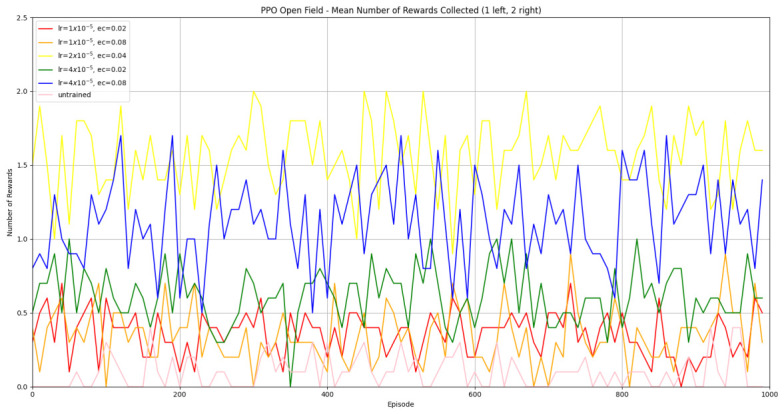
Average number of rewards obtained by open-field-trained PPO at each hyperparameter weighting.

Similarly to A-Learning, the reward-tunnel and open-field PPO agents overfit to the rewarding policy that was initially discovered. However, in this case, the policy was developed during training. PPO agents trained in the reward tunnel had a tendency to move directly forward along the top of the dividing wall and collide with the arena boundary. During training, these actions would have provided rewarding feedback. The lack of rewarding feedback during testing meant that a novel policy for interacting with the environment had to be developed. The expression of exploratory actions led to the eventual detection and obtaining of a reward (see Figure 17). The stochastic nature of the exploration actions resulted in varied mean reward values across hyperparameter weightings, but low standard deviations within each of these weightings (see Figure 18 and Table 4). However, the average number of rewards collected during testing did not stabilise during testing (see Figure 19), suggesting that PPO did not modify its policies to the test environment. The left-hand bias in the highest performing reward-tunnel PPO agent resulted in a much higher average reward being obtained when the left-hand pit contained 2 rewards (see Table 2) as opposed to the right-hand pit (see Table 1). PPO agents trained in the open field had a tendency to navigate the environment in a spiralling manner. In the open field, this continual spiralling would eventually lead to the reward being obtained, but was not effective in the test environment due to the distance at which the agents begin each episode from the rewards (see Figure 20). Similarly to the reward-tunnel-trained version of PPO, varied mean reward values between hyperparameter weightings with low standard deviations were observed (see Figure 21). A lack of stabilisation regarding the average number of rewards collected during testing was also observed (see Figure 22). The right-hand bias in the highest performing open-field PPO agent resulted in a far higher average reward being obtained when the right-hand pit contained two rewards (see Table 1) as opposed to the left-hand pit (see Table 2). The limited success of the trained PPO agents is a result of overfitting to the rewarding policy that was initially discovered, which may be explained in part by PPO’s policy update mechanisms utilising min (see Equation (5)) and max (see Equation (6)) terms. A-Learning’s policy update mechanism (see Equation (2)), by comparison, does not contain a min or max term. Although this exposes A-Learning’s policy updates to greater instability, the heavier policy updates put A-Learning in an advantageous position to more readily adapt to higher-reward policies when discovered. Unlike the reward-tunnel and open-field PPO agents, A-Learning did not show a bias toward entering one pit over another, likely balancing A-Learning’s exploration actions more equally between both pits. In contrast to the reward-tunnel and open-field PPO, the more balanced exploration of both pits resulted in A-Learning’s performance not significantly differing between the two experimental layouts.

#### 3.1.3. Untrained PPO

**Figure 23:**
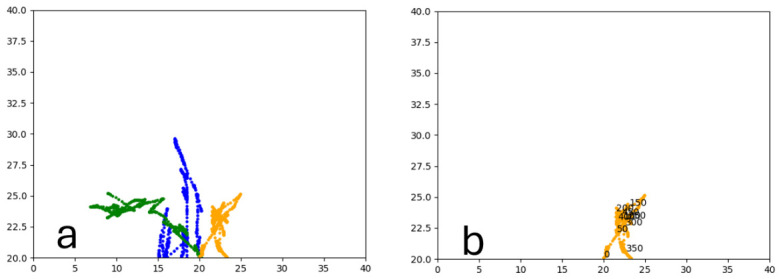
An overview of the ineffective exploration policies expressed by the untrained PPO agent in the 1 left, 2 right experimental layout. Panel (**a**) displays the untrained PPO agent’s exploration policy at episodes 10 (blue), 100 (green), and 1000 (orange). In all 3 episodes, an untrained PPO explored a localised area around the starting point. In some episodes, such as episode 100, for example, an untrained PPO would navigate further away from the start location. However, these actions did not reliably lead the untrained PPO to discover the rewards. The increasing number of episodes with negative feedback discouraged PPO from exploring in a forward direction. Panel (**b**) displays the movement path of the untrained PPO agent at episode 1000, with every 50th timestep numbered.

Untrained PPO obtained the lowest average reward of all agents in both experimental conditions (see Table 1 and Table 2). This is the result of the untrained PPO agent having an ineffective exploration policy (see Figure 23). Obtaining an average reward value that is close to the maximum negative feedback value (−1) suggests that the untrained PPO received too little rewarding feedback to develop an effective policy. This finding aligns with the findings of Crosby et al. (2019), Johnson and Weitzenfeld (2025), and Voudouris et al. (2025) that PPO requires a rich, comprehensive, and detailed training dataset to efficiently pass emulations of cognitive psychology experiments in AAI.

### 3.2. Experiment 2

**Figure 24:**
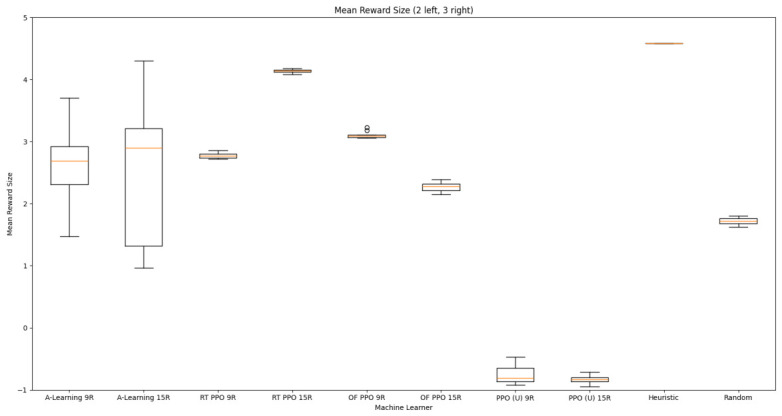
Average reward size obtained for each agent in the 2 left, 3 right experimental layout when receiving 9 (9R) or 15 (15R) raycasts. The mean value acquired by each agent is represented by an orange line. Hyperparameter weightings for the A-learning, reward tunnel (RT) and open field (OF) PPO agents are the same as the highest-performing hyperparameter weightings from Table 1. Results for the untrained PPO (PPO-U), heuristic, and random action agents are also provided.

**Figure 25:**
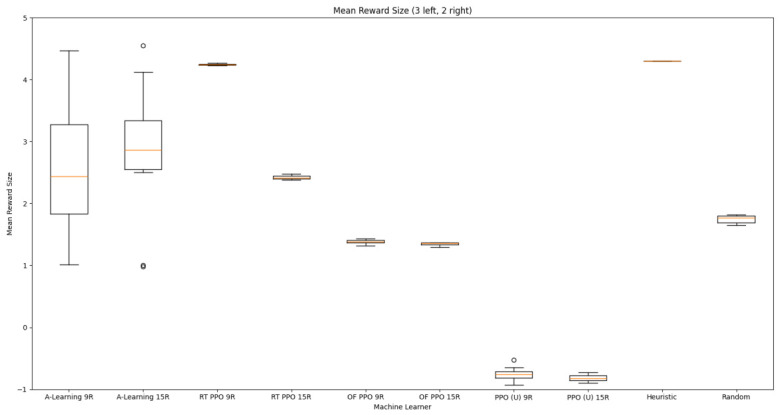
Average reward size obtained for each agent in the 3 left, 2 right experimental layout when receiving 9 (9R) or 15 (15R) raycasts. The mean value acquired by each agent is represented by an orange line. Hyperparameter weightings for the A-learning, reward tunnel (RT) and open field (OF) PPO agents are the same as the highest-performing hyperparameter weightings from Table 1. Results for the untrained PPO (PPO-U), heuristic, and random action agents are also provided.

**Figure 26:**
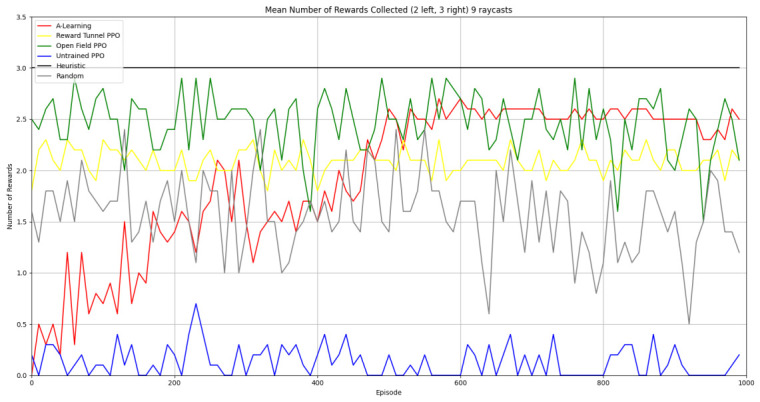
Average number of rewards obtained at each episode in the 2 left, 3 right experimental layout with 9 raycast inputs. Hyperparameter weightings for the A-learning, reward-tunnel and open-field PPO agents are the same as the highest-performing hyperparameter weightings from Table 1. Results for the untrained PPO, heuristic, and random action agents are also provided.

**Figure 27:**
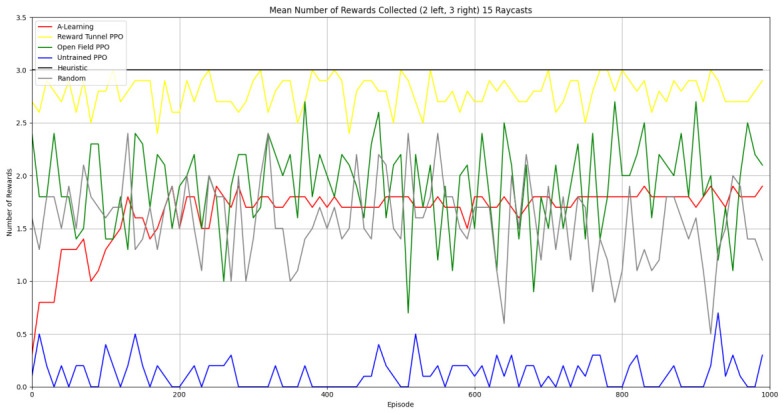
Average number of rewards obtained at each episode in the 2 left, 3 right experimental layout with 15 raycast inputs. Hyperparameter weightings for the A-learning, reward-tunnel and open-field PPO agents are the same as the highest-performing hyperparameter weightings from Table 1. Results for the untrained PPO, heuristic, and random action agents are also provided.

**Figure 28:**
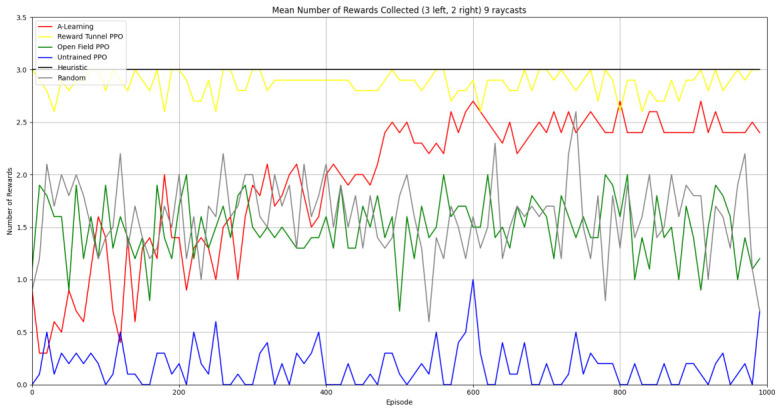
Average number of rewards obtained at each episode in the 3 left, 2 right experimental layout with 9 raycast inputs. Hyperparameter weightings for the A-learning, reward-tunnel and open-field PPO agents are the same as the highest-performing hyperparameter weightings from Table 1. Results for the untrained PPO, heuristic, and random action agents are also provided.

**Figure 29:**
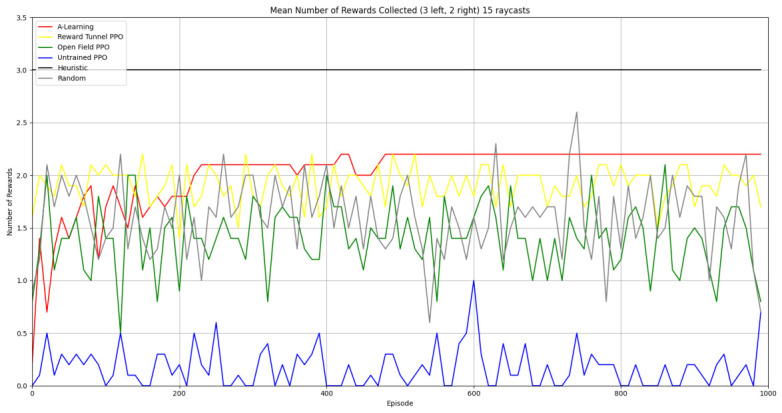
Average number of rewards obtained at each episode in the 3 left, 2 right experimental layout with 15 raycast inputs. Hyperparameter weightings for the A-learning, reward-tunnel and open-field PPO agents are the same as the highest-performing hyperparameter weightings from Table 1. Results for the untrained PPO, heuristic, and random action agents are also provided.

Based on the findings of experiment 1, the A-Learning and PPO agents with the highest-performing hyperparameter weightings were tested in a follow-up experiment with a larger number of rewards (see Figure 7). Two versions of each machine learning agent were tested. These are the original machine learning agents that receive 9 rays from the environment at each timestep, and a second version of each agent that receives 15 rays from the environment at each timestep. Statistically significant differences were observed between the models in the reward layouts of 2 left 3 right (F(9,90)=164.49,p<0.0001) and 3 left 2 right (F(9,90)=99.43,p<0.0001). The reinforcement learning agents did not reliably learn to select the larger reward option in both experimental layouts (see Figure 24 and Figure 25, and Table 5). Similarly to experiment 1, the bias for one pit over another was not found for A-Learning when receiving 9 (t(18)=0.24,p=0.82) or 15 (t(18)=0.51,p=0.62) rays, nor for untrained PPO when receiving 9 (t(18)=0.23,p=0.82) or 15 (t(18)=0.29,p=0.76) rays. Bias was observed for the reward-tunnel-trained PPO at both 9 (t(18)=89.05,p<0.0001) and 15 (t(18)=114.98,p<0.0001) rays, as well as the open-field-trained PPO at both 9 (t(18)=83.22,p<0.0001) and 15 (t(18)=37.13,p<0.0001) rays. The left-hand bias of the reward tunnel-trained PPO from experiment 1 was observed here, as was the right-hand bias of the open field-trained PPO. However, the 15-ray versions of the reward tunnel and open-field-trained PPO expressed a right- and left-hand bias, respectively. The bias of both trained PPO agents, therefore, switched between the 9- and 15-ray versions. This suggests that the reward tunnel and open-field training environments do not inherently favour one direction over another, and left- or right-handed bias likely emerged in response to the initially rewarded policy each agent experienced during training. If that policy included moving left, for example, the agent would reinforce actions with a left-sided bias, which were subsequently observed during testing. This can be observed in relation to the average number of rewards collected by the agents during testing. A-Learning developed a comparatively stable policy that plateaued during testing. The average number of rewards collected by both trained and untrained PPO remained stochastic throughout testing across all experimental conditions (see Figure 26, Figure 27, Figure 28 and Figure 29). Further support for a lack of numerosity capabilities in A-Learning and PPO can be seen when comparing the 9- and 15-ray versions of each agent. For A-Learning, receiving 9 or 15 rays did not significantly differ the agent’s performance in either the 2 left, 3 right (t(18)=0.26,p=0.80), or the 3 left, 2 right (t(18)=0.50,p=0.62) experimental layout. Similarly, the performance of the untrained PPO agent was not significantly different in the 2 left, 3 right (t(18)=1.55,p=0.15) or 3 left, 2 right (t(18)=1.45,p=0.17) experimental layouts. However, significant differences were observed for the trained PPO agents. In the case of the reward tunnel-trained PPO agent, the 15-ray version outperformed the 9-ray version in the 2 left, 3 right experimental layout (t(18)=71.79,p<0.0001). This is due to the 15-ray version having a right-hand bias, whilst the 9-ray version had a left-hand bias. Indeed, the results are swapped in the 3 left, 2 right experimental layout, where the 9-ray version outperformed the 15-ray version (t(18)=156.51,p<0.0001). Similar results were also observed for the open-field-trained PPO agent. In the 2 left, 3 right experimental layout, the right-hand-biased 9-ray version outperformed the left-hand-biased 15-ray version (t(18)=61.53,p<0.0001). In the 3 left, 2 right experimental layout, the 15-ray version outperformed the 9-ray version (t(18)=57.08,p<0.0001). This finding corroborates those of experiment 1 and suggests that receiving 9 or 15 rays does not affect the numerosity capabilities of the A-Learning or untrained PPO agents. In the case of the trained PPO agents, biases developed during training eclipsed any potential benefits that the 15-ray versions may have provided. Despite receiving only 9 ray inputs per timestep, the heuristic agent outperformed all other agents and achieved three rewards in 100% of episodes across both experimental layouts. Although the heuristic agent was not capable of responding to all three rewards simultaneously and lacked memory to record the locations of previously detected rewards, the pit with the larger number of rewards provided more inputs over successive timesteps. This caused the pit with the larger number of rewards to have a greater ‘pull’ on the heuristic agent. As in experiment 1, the heuristic agent again produced a higher average reward value when the right-hand pit contained the larger amount of rewards as opposed to the left-hand pit.

### 3.3. Experiment 3

**Figure 30:**
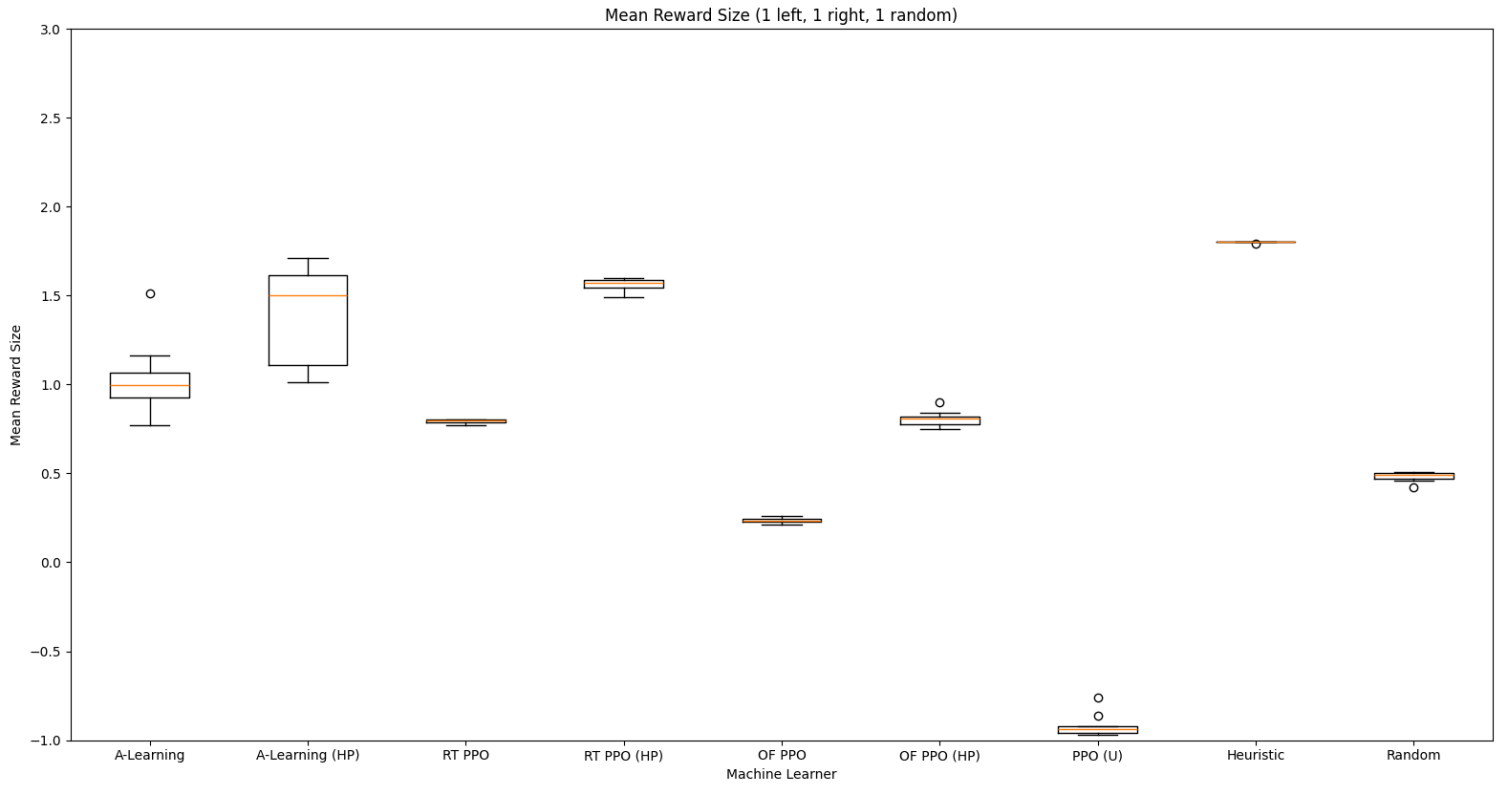
Average reward size obtained for each agent in the 1 left, 1 right, 1 random experimental layout. The mean value acquired by each agent is represented by an orange line. Hyperparameter weightings for the highest-performing (HP) A-learning, reward-tunnel (RT), and open-field (OF) PPO agents are the same as the highest-performing hyperparameter weightings from Table 6. Results for the untrained PPO (PPO-U), heuristic, and random action agents are also provided.

**Figure 31:**
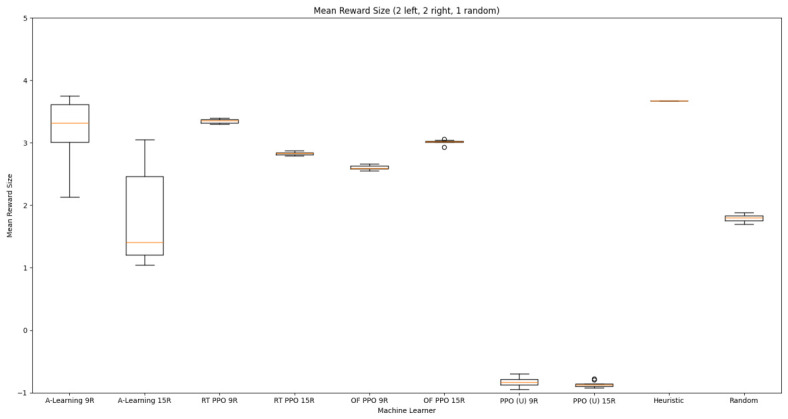
Average reward size obtained for each agent in the 2 left, 2 right, 1 random experimental layout when receiving 9 (9R) or 15 (15R) raycasts. The mean value acquired by each agent is represented by an orange line. Hyperparameter weightings for the highest performing (HP) A-learning, reward tunnel (RT) and open field (OF) PPO agents are the same as the highest-performing hyperparameter weightings from Table 6. Results for the untrained PPO (PPO-U), heuristic, and random action agents are also provided.

**Figure 32:**
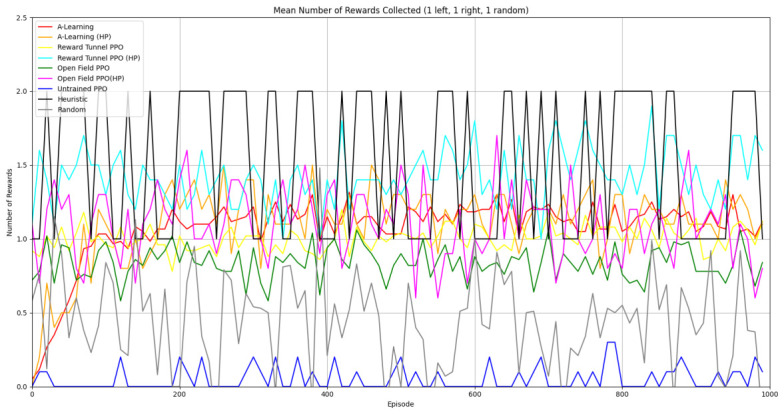
Average number of rewards obtained at each episode in the 1 left, 1 right, 1 random experimental layout. Hyperparameter weightings for the highest-performing (HP) A-learning, reward-tunnel, and open-field PPO agents are the same as the highest-performing hyperparameter weightings from Table 6. Results for the untrained PPO, heuristic, and random action agents are also provided.

**Figure 33:**
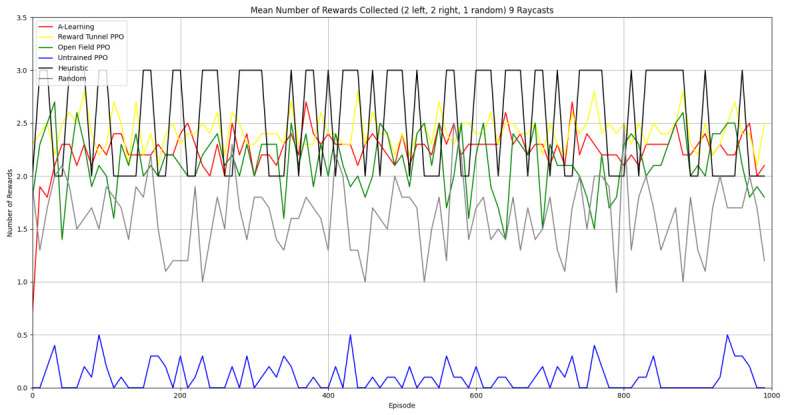
Average number of rewards obtained at each episode in the 2 left, 2 right, 1 random experimental layout with 9 raycasts. Hyperparameter weightings for the A-learning, reward-tunnel and open-field PPO agents are the same as the highest-performing hyperparameter weightings from Table 6. Results for the untrained PPO, heuristic, and random action agents are also provided.

**Figure 34:**
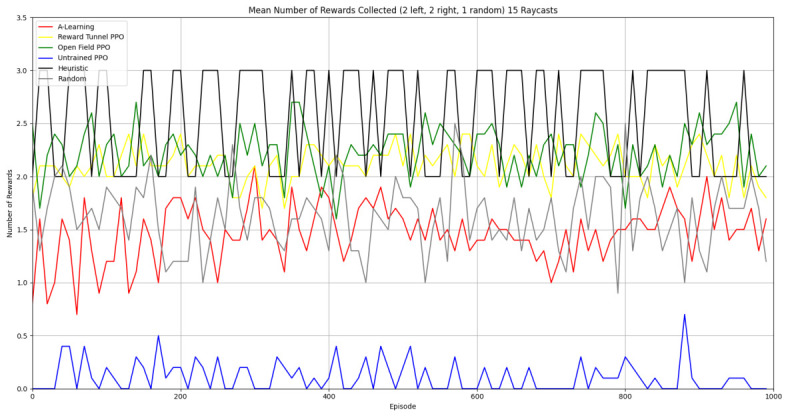
Average number of rewards obtained at each episode in the 2 left, 2 right, 1 random experimental layout with 15 raycasts. Hyperparameter weightings for the A-learning, reward-tunnel and open-field PPO agents are the same as the highest-performing hyperparameter weightings from Table 6. Results for the untrained PPO, heuristic, and random action agents are also provided.

Experiment 3 introduced additional noise into the environment by having 1 reward spawn randomly in the left- or right-hand pit in each episode (see Figure 8 and Figure 9). This prevented the agents from potentially passing the experiments through route learning behaviours. Statistically significant differences between models were observed in both the 1 left, 1 right, 1 random (F(5,54)=2806.75,p<0.0001) and 2 left, 2 right, 1 random (F(9,90)=281.17,p<0.0001) reward layouts. However, neither A-Learning nor PPO reliably passed either layout. The heuristic agent obtained a higher average score than the other agents (see Figure 30 and Figure 31, and Table 6), but unlike experiments 1 and 2, did not obtain the largest quantity of rewards in every episode. This is because the reward that begins each episode in a random location would occasionally occlude other rewards, causing the heuristic agent to prematurely enter one of the lower branches of the behaviour tree. Introducing a bespoke search function to the heuristic agent, or requiring the agent not to leave the diving barrier until a specified number of rewards is detected in a single pit, would likely have enabled the heuristic agent to perform the same as experiments 1 and 2. However, these additional specifications would move the heuristic agent beyond the scope of simple numerosity-embedded capabilities towards a representation of bespoke solutions for each experimental layout. The heuristic agent was therefore not modified in experiments 1 and 2 to avoid disturbing its representation of simple numerosity capabilities. Similarly to experiments 1 and 2, the average number of rewards acquired by A-Learning was more stable than PPO during testing when receiving 9 rays (see Figure 32 and Figure 33). However, a greater instability was observed when A-Learning received 15 rays (see Figure 34).

#### 3.3.1. A-Learning

**Figure 35:**
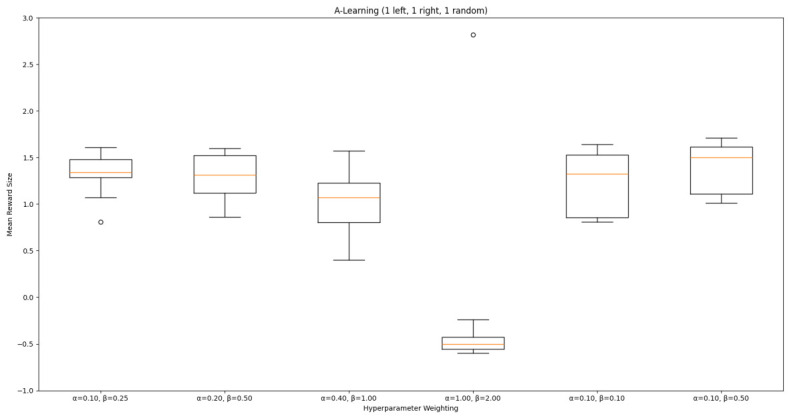
Average reward size obtained by A-Learning at each hyperparameter weighting. The mean value acquired by each agent is represented by an orange line.

**Figure 36:**
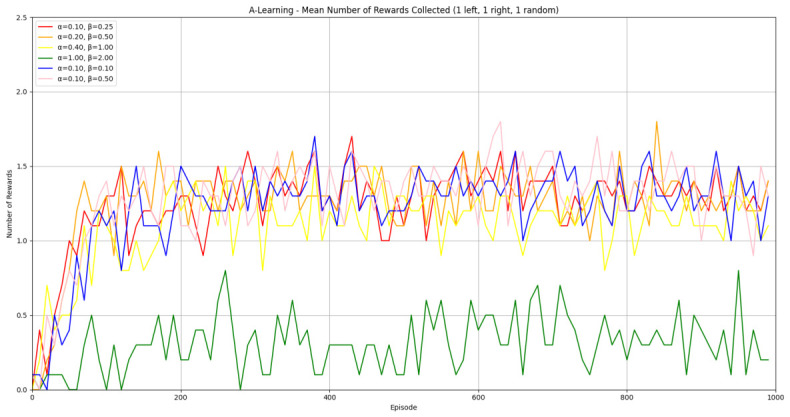
Average number of rewards obtained by A-Learning at each hyperparameter weighting.

In experiment 1, A-Learning with a hyperparameter weighting of α=1.00,β=2.00 obtained the highest-average reward value (see Table 3). Here, the same hyperparameter weighting obtained the lowest-average reward value (see Figure 35) and the number of rewards during testing (see Figure 36). This difference in results is likely due to the more stochastic nature of experiment 3. In experiment 1, rewards are always delivered at the same location in each episode. Therefore, the high α and β values were advantageous, as the agent would frequently explore the environment and update its policy weightings more frequently when a more rewarding policy was discovered. However, the greater variance here caused the A-Learning agent to update its policy weightings too heavily to novel reward locations. Here, the highest-performing A-Learning agent had hyperparameter weightings of α=0.10,β=0.50. This weighting provided comparatively light updates to novel experiences, whilst still expressing a relatively high number of exploratory actions. These weightings facilitated more stable policy updates, whilst exploring the environment frequently enough to discover the reward that begins each episode in a random location. This finding provides further evidence for the sensitivity of A-Learning’s hyperparameter weightings to varied experimental layouts. A significant difference was found in the 2 left, 2 right, 1 random experimental layout, where the 9-ray version significantly outperformed the 15-ray version (t(18)=4.61,p=0.0003). Further suggesting that A-Learning is acting in the environment in terms of reinforced movement policies, which may favour the less complex 9-ray version, as opposed to deliberate planning and numerosity capabilities, which would favour the 15-ray version.

#### 3.3.2. Trained PPO

**Figure 37:**
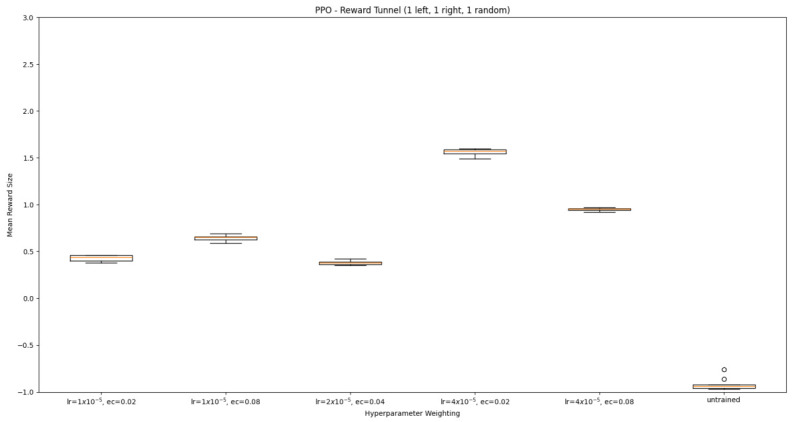
Average reward size obtained by reward-tunnel-trained PPO at each hyperparameter weighting. The mean value acquired by each agent is represented by an orange line.

**Figure 38:**
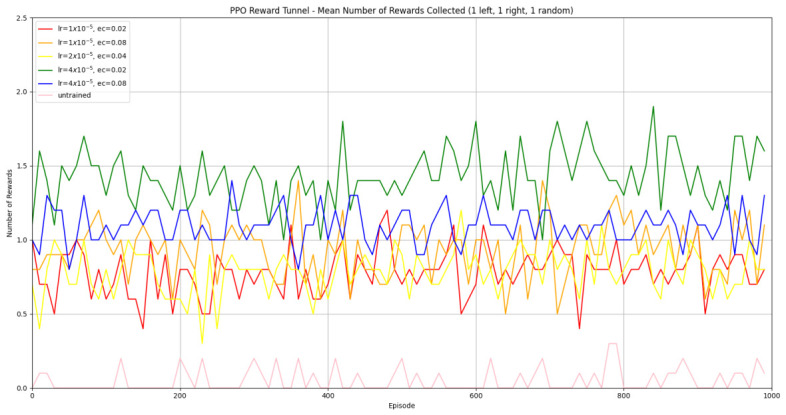
Average number of rewards obtained by reward-tunnel-trained PPO at each hyperparameter weighting.

**Figure 39:**
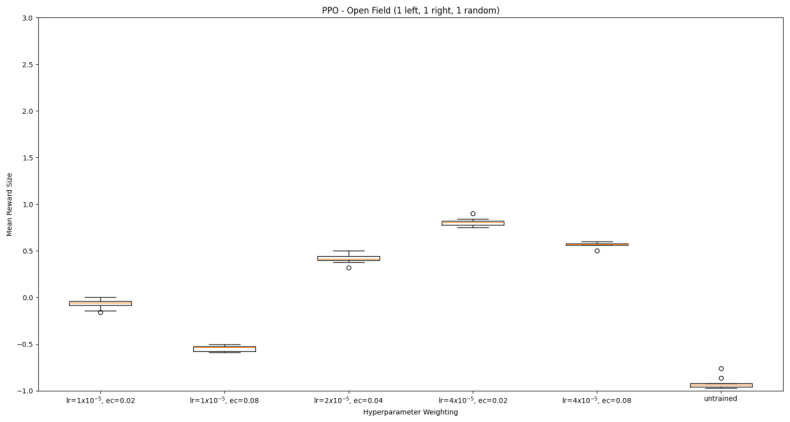
Average reward size obtained by open-field-trained PPO at each hyperparameter weighting. The mean value acquired by each agent is represented by an orange line.

**Figure 40:**
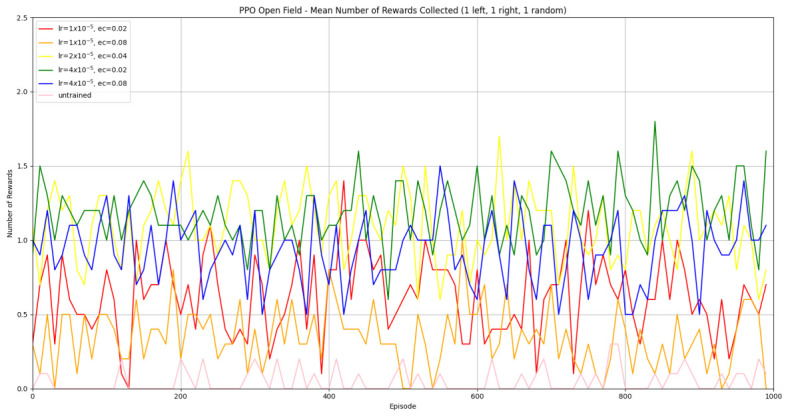
Average number of rewards obtained by open-field-trained PPO at each hyperparameter weighting.

Similarly to experiment 1, the highest performing reward tunnel trained hyperparameter weighting was $lr=4\times 10^{-5},ec=0.02$ (see Figure 37 and Figure 38). The open-field-trained PPO agent also obtained the highest average performance at this weighting (see Figure 39 and Figure 40). This suggests that the hyperparameter weightings of PPO have a greater stability to novel contexts than A-Learning, which would align with the policy update functions of A-Learning (see Equation (2)) not containing the more conservative min (see Equation (5)) and max (see Equation (6)) terms in PPO’s policy updates. Similarly to experiment 2, significant differences were observed between the 9- and 15-ray versions of each agent in the 2 left, 2 right,1 experimental layout. Here, the 9-ray version of the reward-tunnel-trained PPO agent outperformed the 15-ray version (t(18)=36.47,p=0.0001), whilst the 15-ray version of the open-field-trained PPO agent outperformed the 9-ray version (t(18)=26.60,p<0.0001). However, unlike experiment 2, a left- or right-hand bias does not favour an experimental layout in each iteration. It is possible that, as in A-Learning, the agents are interacting with reinforcement-based movement policies. Here, the less complex 9-ray version may be of benefit to the reward-tunnel PPO agent, while the 15-ray version may be of benefit to the open-field PPO agent. As is the case in experiment 2, biases developed during training overshadowed any potential benefits that the 15-ray version may have provided in terms of greater numerosity capabilities.

#### 3.3.3. Untrained PPO

As in experiments 1 and 2, the untrained PPO agent obtained the lowest average reward value in both experimental conditions (see Table 6), while the 9- and 15-ray versions did not differ significantly from each other in the 2 left, 2 right, 1 random experimental layout (t(18)=1.34,p=0.20). These findings corroborate the results of experiments 1 and 2 that PPO requires training prior to testing, as the lack of reinforcing feedback during testing results in an increasingly restricted exploration policy (see Figure 23).

## 4. Discussion

The increasing power and complexity of AI systems have contributed to an increased interest in evaluating these systems along more psychologically inspired lines (Capel & Brereton, 2023; Lake et al., 2017; Shneiderman, 2022). In particular, researchers such as Burden et al. (2023), Burnell et al. (2023), Hernández-Orallo (2020) and Rutar et al. (2025) recommend that multifaceted, domain-general evaluation methods inspired by the psychological literature be adopted. To support these recommendations, we tested the numerosity capabilities of a reinforcement learning model, A-Learning, in AAI. This is because A-Learning has been predicted to have numerosity capabilities (Lind & Vinken, 2021) but has not been tested in an embodied environment that is emulative of real-world numerosity experiments with human and non-human animals.

### 4.1. Summary of Findings

Both A-Learning and trained PPO performed unreliably in the three experiments. Although both agents occasionally developed effective policies, this did not hold consistently across the three experiments. A-Learning, for example, overfitted to the initially rewarding policy that was discovered during testing. Hyperparameter instability was also observed, where α=1.00,β=2.00 obtained the highest average reward value in experiment 1 (see Figure 14), but the lowest in experiment 3 (see Figure 35). Consequently, A-Learning did not reliably select the larger of the two reward options as Lind and Vinken (2021) predicted. Trained PPO, by comparison, did not display the same hyperparameter instability across the experiments but did overfit to the policy developed during training. Both the reward tunnel and open field agents displayed a bias for entering one of the pits over another. This bias depended on the direction the agent was travelling when it received rewarding feedback during training. The untrained version of PPO did not explore the environment effectively enough to discover and reinforce a policy that reliably obtained rewards. This finding supports previous research by Crosby et al. (2019) and Voudouris et al. (2025), who found that a rich, comprehensive, and detailed training dataset is required for PPO to perform well in AAI experiments. Without this, PPO is not likely to perform well in testing.

### 4.2. Discussion and Implications

The lack of replication between our experimental findings and Lind and Vinken (2021)’s is a result of variations in experimental design. Our experiments were conducted in AAI, which is an embodied virtual space, while Lind and Vinken (2021)’s were conducted in a scripted (i.e., text-based) environment known as the Animal Learning Simulator. The greater environmental complexity of our experiment required the agents to navigate the environment via timesteps rather than transitioning between predefined state spaces. This greater variance removes the binary classification by Lind and Vinken (2021) of the reward as more or less, and increases the number of states and policies in which rewards can be discovered. Indeed, our experiment introduces 0 value state spaces to the numerosity experiment, which were not included in Lind and Vinken (2021)’s experiment. In the Animal Learning Simulator, A-Learning was consistently rewarded for exploratory actions that moved it from the less rewarding to the more rewarding state space. Here, A-Learning is required to not only express exploratory actions, but also to direct those actions towards a specific location in the environment, as animals are required to do in real-world numerosity research. Consequently, A-Learning was more likely to discover and reinforce a policy that did not yield the largest reward value. If a reward is not discovered when exploring, rewarding feedback will not be provided. This may discourage further exploration in future episodes. This is particularly the case if an alternative policy that results in a reward of some value being obtained already exists. The difference in findings between ours and Lind and Vinken (2021)’s experiments evidences a suggestion by Voudouris et al. (2024) that predicting behaviours via transitory state spaces removes much of the explanatory relevance to the cognitive capability being assessed. By embodying A-Learning and PPO into a virtual avatar in AAI, we hope to have returned some of this explanatory relevance. Our finding is that the greater variance and stochastic nature of AAI resulted in both models not performing reliably. Occasionally, but not consistently, developing a policy that led to the larger reward option being obtained. Further developments to both the A-Learning and PPO models will likely be required if numerosity capabilities are to be reliably expressed. We will now discuss potential modifications to the A-Learning and PPO models, which may assist in the development of numerosity capabilities.

#### 4.2.1. A-Learning

In the context of A-Learning, Equations (1) and (2) provide the mechanisms for stimulus weighting and value assignment to stimulus-response pairings, respectively. Although Equation (1) assigns a value to stimuli such that different stimulus inputs will be more heavily weighted than others, this does not allow A-Learning to discriminate between stimulus inputs of the same classification in distinct environmental contexts. Here, therefore, the A-Learning agent is capable of heavily weighing reward inputs, but not weighing the value of the individual reward inputs from one pit more heavily than the reward inputs in the other pit. Further modifications to Equation (1) that allow context-dependent modifications in the weighing of stimuli may help address this limitation. If, in the 1 left, 2 right experimental layout, for example, Equation (1) also factored in the relative distances of the stimulus objects from each other, the rewards in the right-hand pit would be assigned a higher weight than the reward in the left-hand pit. This approach may also address a limitation of Equation (2), that the stimulus-response pairings are updated independently of other stimulus-response pairings. If, for example, the A-Learning agent reinforces a stimulus-response pairing to obtain one reward in the right-hand pit, this does not reinforce a stimulus-response pairing to obtain the remaining reward in the right-hand pit, as demonstrated in Figure 16. The prior suggestion to introduce an additional factor regarding the relative distances of stimulus objects from one another may help address this limitation. Another approach may be the introduction of a mechanism by which non-identical stimulus-response pairings influence the value of one another when updated; similarly to higher-order conditioning models in the cognitive psychology literature (Holland & Rescorla, 1975; Honey & Dwyer, 2022). In the context of this experiment, such a mechanism would enable the updating of a stimulus-response pairing to have a secondary influence on policies for obtaining other rewards. The design and introduction of these suggested modifications will be non-trivial, however, and will likely require validation through extensive testing before being considered a beneficial amendment to the existing A-Learning model.

#### 4.2.2. PPO

The PPO model will likely require further development if numerosity capabilities are to be reliably expressed. In this experiment, PPO did not reliably display numerosity capabilities due to overfitting to the policy developed during training. If not trained prior to testing, the PPO did not explore the environment effectively enough to discover and reinforce a rewarding policy. These findings may be explained in part by the conservative parameter updates shown in Equations (5) and (6). In this way, updates to existing policy weightings are minimised to avoid dramatic changes to an existing policy and encourage monotonic improvements in performance (Schulman et al., 2017). However, this approach is likely to have inhibited PPO from adapting its parameter weightings to a novel reward signal. If, for example, PPO initially discovers and reinforces a policy for obtaining a reward in the left-hand pit, the conservative policy updates from exploratory actions in which the rewards in the right-hand pit are obtained are likely to lack the necessary magnitude to modify the model’s behaviour. Although the reward tunnel and open field agents both adapted their policies occasionally (see Figure 17 and Figure 20, respectively), this occurred neither predictably nor reliably across the 3 experiments. The existence of an initial policy which provides a positive reward signal may therefore inhibit the reinforcement of a novel, larger reward signal. This occurrence would be similar to the blocking phenomena observed in associative learning research, whereby a rewarding stimulus-response association inhibits the reinforcement and subsequent learning of a second novel stimulus-response mechanism (Haselgrove, 2016; J. Liu et al., 2023). Modifications to Equations (5) and (6) to increase the magnitude of updates to larger reward signals may help to discourage these overly conservative policy updates and potential blocking-like phenomena. Another approach may be to increase the amount of exploration performed by the model. Greater exploration of the environmental space may increase the frequency with which additional rewarding policies are discovered. This, in turn, may mitigate against overfitting to an initially rewarding policy. Additionally, the previously suggested introduction of a mechanism that allows state-action pairings to influence one another may also be beneficial to the PPO model. If introduced, the updating of a state-action pair to obtain one of the rewards may also update similar but non-identical state-action pairings for obtaining other rewards. As is the case with A-Learning, however, designing and introducing these suggested modifications will be non-trivial and will likely require validation through extensive testing. The PPO agents in our experiment were trained for a much shorter period than in the experiments by Voudouris et al. (2025). In their experiments, PPO was trained for 2 million timesteps, as opposed to 10,000 in our experiment. Our findings, therefore, suggest that A-Learning is capable of outperforming PPO within the computational budget provided here. However, this difference in timesteps is not likely to have inhibited performance, as additional training is likely to have caused greater overfitting to the policy learnt during training. Indeed, the top-performing models in Beyret et al. (2019)’s numerosity experiments also passed the tests unreliably. The competition-winning model, for example, was a PPO agent trained for 70 million timesteps. Our findings, therefore, suggest that training a PPO agent to navigate toward rewarding stimuli is not efficient for passing tests of numerosity. Instead, PPO should be provided with more varied training environments that provide a richer representation of the capabilities necessary for numerosity to emerge.

### 4.3. Limitations and Future Research

Our experiment has several limitations. Firstly, we only provided raycasting as the sensory mechanism for the embodied agents. Raycasting is the preferred sensory mechanism, as discrete value representations of objects in the environment provide a closer emulation of the experimental design by Lind and Vinken (2021). However, running a version of the numerosity experiment in which agents received pixel inputs would broaden our understanding of the numerosity capabilities of A-Learning and PPO in a context more emulative of the visual mechanisms used by human and non-human animals to discern quantity. Based on research by Cardoso et al. (2023), we predict that A-Learning and PPO would perform less reliably with pixel inputs than with raycast inputs. This will likely be due to greater variance in object recognition and stimulus classification that is expected to emerge with continuous pixel inputs from computer vision, as opposed to discrete value inputs from raycasts. Secondly, A-Learning and PPO are not the only reinforcement learning models. The soft-actor critic (SAC) model, for example, is another reinforcement learning model which aims to maximise both the expected return and the expected exploration of a learnt policy (Haarnoja et al., 2018). Although predictions of the numerosity capabilities of SAC are lacking, previous spatial navigation research has found that SAC is more efficient than PPO in discovering rewarding policy behaviours in continuous (Deowan et al., 2025; Elallid et al., 2024; Y. Liu et al., 2024) but not discrete action spaces (Asad et al., 2025; Zhu et al., 2021). Because the agents in our experiment navigate the environment using discrete actions, PPO was selected over SAC. However, repeating our experiment with SAC agents that interact with the environment using a continuous action space may be a promising avenue for future research. If, for example, the SAC model began to learn a policy for entering the left-hand pit, the aim of SAC to maximise exploration may lead to the SAC model entering the right-hand pit, encouraging a more balanced exploration of both pits than A-Learning or PPO. Testing SAC and other trust region policy optimisation models beyond PPO would also improve the generalisability of our findings. Third, future research may be interested in more in-depth research on the information processing mechanisms and architecture of the agents (Jarvenpaa et al., 1985). By better understanding how the agents perceive and respond to inputs in more nuanced ways, researchers may be better equipped to modify the architectures of the A-Learning and PPO agents to improve the numerosity capabilities of both models in future embodiment experiments. Finally, future research would likely benefit from direct comparisons with human and non-human participants, as Voudouris et al. (2022) and Cardoso et al. (2023) did, respectively. Such research would provide a rich dataset on the performance capabilities and behavioural tendencies of organisms with numerosity capabilities, especially when compared with the heuristic agent used here as a representation of embedded numerosity capabilities. This richer dataset would provide a more in-depth point of reference into the capabilities that A-Learning and PPO appear to be lacking here.

## 5. Conclusions

In summary, this study investigated the numerosity capabilities of two reinforcement learning models, A-Learning and PPO, in an embodied virtual environment, AAI. In contrast to previous predictions by Lind and Vinken (2021) we found that A-Learning did not reliably express numerosity capabilities. This is because the first rewarding behaviour discovered would be reinforced more readily than alternative, possibly more rewarding behaviours discovered in later episodes. The trained versions of PPO overfitted to the policy learnt during training and did not reliably learn to adapt this policy to obtain the larger reward option during testing. The untrained version of PPO did not explore the environment effectively enough to reliably discover and reinforce a policy that would obtain rewards. These findings suggest that the greater environmental complexity of both AAI and the perceptual mechanisms from embodiment resulted in A-Learning and PPO not reliably learning numerosity capabilities within the computational budget of our experiments. As human and non-human animals are capable of displaying far more sophisticated forms of numerosity, in more complex real-world environments (Brannon, 2005; Cicchini et al., 2016; Nieder, 2021), this suggests that there are fundamental cognitive processing mechanisms involved with numerosity perception and understanding that neither A-Learning nor PPO can fully emulate without greater additional training and support (e.g., supervised and/or model-based learning) mechanisms than is provided here. In conclusion, predicting the capabilities of machine learning systems is non-trivial, especially when machine learning systems are embodied. This is because variations in, and perceptions of, the environment introduce additional confounds for machine learning systems to account for. Examples of these confounding factors in more real-world terms may include situations that require reliable identification of quantity, such as the number of pedestrians crossing a road for self-driving vehicles or object identification and retrieval in the case of automated delivery vehicles. To overcome these challenges, future researchers will likely benefit from recommendations to assess the capabilities of machine learning systems in a multifaceted, competency-oriented manner that is emulative of assessment methods used in psychological research (Burden et al., 2023; Burnell et al., 2022; Hernández-Orallo, 2020; Rutar et al., 2025; Voudouris et al., 2024).

## Figures and Tables

**Table 1:** Average reward size obtained by each agent in the 1 left, 2 right experimental layout. Results are provided for the mean and highest-performing (HP) hyperparameter weightings of A-Learning (α=1.0,β=2.0), and the reward-tunnel ($lr=4\times 10^{-5},EC=0.02$) and open-field ($lr=2\times 10^{-5},EC=0.04$) PPO agents.

Machine Learner	Mean	Standard Deviation
A-Learning	1.95	0.35
A-Learning (HP)	2.32	0.86
Reward-tunnel PPO	0.52	0.01
Reward-tunnel PPO (HP)	0.88	0.02
Open-field PPO	0.23	0.01
Open-field PPO (HP)	1.30	0.03
Untrained PPO	−0.87	0.09
Heuristic	2.93	0
Random	0.46	0.05

**Table 2:** Average reward size obtained by each agent in the 2 left, 1 right experimental layout. Hyperparameter weightings for the A-learning, reward-tunnel and open-field PPO agents are the same as the highest-performing hyperparameter weightings from Table 1.

Machine Learner	Mean	Standard Deviation
A-Learning	2.44	0.48
Reward-tunnel PPO	2.26	0.01
Open-field PPO	−0.11	0.03
Untrained PPO	−0.85	0.07
Heuristic	2.34	0
Random	0.48	0.04

**Table 3:** Means and standard deviations of A-Learning at each hyperparameter weighting.

A-Learning HP Weighting	Reward Layout	Mean	Standard Deviation
α=0.10,β=0.25	1 left, 2 right	1.78	1.01
α=0.20,β=0.50	1 left, 2 right	2.20	0.77
α=0.40,β=1.00	1 left, 2 right	1.98	0.95
α=1.00,β=2.00	1 left, 2 right	2.32	0.86
α=1.00,β=2.00	2 left, 1 right	2.44	0.48
α=0.10,β=0.10	1 left, 2 right	1.56	1.01
α=0.10,β=0.50	1 left, 2 right	1.89	0.93

**Table 4:** Means and standard deviations of PPO at each hyperparameter weighting.

PPO HP Weighting	Training	Reward Layout	Mean	Standard Deviation
$lr=1\times 10^{-5},EC=0.02$	Reward Tunnel	1 left, 2 right	0.52	0.02
$lr=1\times 10^{-5},EC=0.08$	Reward Tunnel	1 left, 2 right	0.38	0.04
$lr=2\times 10^{-5},EC=0.04$	Reward Tunnel	1 left, 2 right	0.06	0.04
$lr=4\times 10^{-5},EC=0.02$	Reward Tunnel	1 left, 2 right	0.88	0.02
$lr=4\times 10^{-5},EC=0.02$	Reward Tunnel	2 left, 1 right	2.26	0.01
$lr=4\times 10^{-5},EC=0.08$	Reward Tunnel	1 left, 2 right	0.79	0.04
$lr=1\times 10^{-5},EC=0.02$	Open Field	1 left, 2 right	−0.53	0.02
$lr=1\times 10^{-5},EC=0.08$	Open Field	1 left, 2 right	−0.46	0.03
$lr=2\times 10^{-5},EC=0.04$	Open Field	1 left, 2 right	1.30	0.03
$lr=2\times 10^{-5},EC=0.04$	Open Field	2 left, 1 right	−0.11	0.03
$lr=4\times 10^{-5},EC=0.02$	Open Field	1 left, 2 right	−0.05	0.03
$lr=4\times 10^{-5},EC=0.08$	Open Field	1 left, 2 right	0.90	0.04
$lr=2\times 10^{-5},EC=0.04$	Untrained	1 left, 2 right	−0.87	0.09
$lr=2\times 10^{-5},EC=0.04$	Untrained	2 left, 1 right	−0.85	0.07

**Table 5:** Average reward size obtained by each agent in the ‘2v3’ experimental layouts (see Figure 7). Hyperparameter weightings for the A-learning, reward-tunnel and open-field PPO agents are the same as the highest-performing hyperparameter weightings from Table 1.

Machine Learner	Raycasts	Reward Layout	Mean	Standard Deviation
A-Learning	9	2 left, 3 right	2.64	0.61
	15	2 left, 3 right	2.53	1.24
Reward-tunnel PPO	9	2 left, 3 right	2.78	0.05
	15	2 left, 3 right	4.14	0.03
Open-field PPO	9	2 left, 3 right	3.11	0.06
	15	2 left, 3 right	1.58	0.05
Untrained PPO	9	2 left, 3 right	−0.75	0.15
	15	2 left, 3 right	−0.83	0.07
Heuristic	9	2 left, 3 right	4.58	0
Random	9	2 left, 3 right	1.72	0.06
A-Learning	9	3 left, 2 right	2.55	1.12
	15	3 left, 2 right	2.80	1.15
Reward-tunnel PPO	9	3 left, 2 right	4.25	0.01
	15	3 left, 2 right	2.42	0.03
Open-field PPO	9	3 left, 2 right	1.38	0.03
	15	3 left, 2 right	2.43	0.05
Untrained PPO	9	3 left, 2 right	−0.76	0.12
	15	3 left, 2 right	−0.82	0.05
Heuristic	9	3 left, 2 right	4.30	0
Random	9	3 left, 2 right	1.75	0.07

**Table 6:** Average reward size obtained by each agent in the ‘1v1(+1 random)’ (see Figure 8) and ‘2v2 (+1 random)’ (see Figure 9) experimental layouts. Results are provided for the mean and highest performing (HP) hyperparameter weightings of A-Learning (α=0.10,β=0.50), and the reward tunnel ($lr=4\times 10^{-5},EC=0.02$) and open field ($lr=4\times 10^{-5},EC=0.02$) PPO agents.

Machine Learner	Raycasts	Reward Layout	Mean	Standard Deviation
A-Learning	9	1 left, 1 right, 1 random	1.02	0.21
A-Learning (HP)	9	1 left, 1 right, 1 random	1.39	0.28
Reward-tunnel PPO	9	1 left, 1 right, 1 random	0.79	0.01
Reward-tunnel PPO (HP)	9	1 left, 1 right, 1 random	1.56	0.03
Open-field PPO	9	1 left, 1 right, 1 random	0.24	0.02
Open-field PPO (HP)	9	1 left, 1 right, 1 random	0.81	0.05
Untrained PPO	9	1 left, 1 right, 1 random	−0.92	0.06
Heuristic	9	1 left, 1 right, 1 random	1.80	0.003
Random	9	1 left, 1 right, 1 random	0.48	0.03
A-Learning (HP)	9	2 left, 2 right, 1 random	3.20	0.54
	15	2 left, 2 right, 1 random	1.78	0.81
Reward-tunnel PPO (HP)	9	2 left, 2 right, 1 random	3.35	0.04
	15	2 left, 2 right, 1 random	2.42	0.03
Open-field PPO (HP)	9	2 left, 2 right, 1 random	2.60	0.04
	15	2 left, 2 right, 1 random	3.02	0.03
Untrained PPO	9	2 left, 2 right, 1 random	−0.83	0.07
	15	2 left, 2 right, 1 random	−0.87	0.05
Heuristic	9	2 left, 2 right, 1 random	3.56	0
Random	9	2 left, 2 right, 1 random	1.80	0.06

## Data Availability

The raw data supporting the conclusions of this article will be made available by the authors on request.
